# Emerging Nanobiochar –Hydrogel Therapeutic Systems: Redox Modulation, Biointerface Interactions, and Critical Gaps in In Vitro Evaluation

**DOI:** 10.3390/gels12080660

**Published:** 2026-07-23

**Authors:** Vidhya Sunil Bhaskarakurup, Leena Thomas, Rawan Abusirdaneh, Dali Vilma Francis, Rema M. Amawi

**Affiliations:** Department of Math & Sciences, Rochester Institute of Technology (RIT), Dubai 345055, United Arab Emirates; vsbcad@rit.edu (V.S.B.); lxtcad@rit.edu (L.T.); raacad@rit.edu (R.A.)

**Keywords:** nanobiochar, hydrogel, wound healing, redox modulation, reactive oxygen species, biointerface interactions, protein corona, cellular signaling, cytocompatibility, regenerative medicine

## Abstract

Nanobiochar has attracted increasing attention as a redox-active carbon nanomaterial with potential applications beyond its traditional roles in environmental remediation and adsorption technologies. When integrated into hydrogel matrices, nanobiochar may provide a unique combination of physicochemical and biological functionalities, including reactive oxygen species (ROS) modulation, antimicrobial activity, high adsorption capacity, and localized therapeutic delivery. Such properties are particularly relevant to emerging wound-healing and regenerative medicine applications; however, the biological mechanisms governing the performance of nanobiochar–hydrogel systems remain poorly understood. Because direct studies on nanobiochar–hydrogel therapeutic systems remain scarce, this review integrates evidence from the limited nanobiochar literature together with evidence from studies on conventional biochar, hydrogel biomaterials, and related carbon nanomaterial to critically evaluate emerging biological mechanisms and identify future research priorities. This review combines bibliometric analysis with mechanistic evaluation to assess the potential of nanobiochar–hydrogel systems as therapeutic biomaterials while highlighting critical knowledge gaps limiting their development. Bibliometric findings reveal that research on biochar–hydrogel composites is dominated by environmental remediation, adsorption processes, and material characterization, whereas investigations addressing biological responses and therapeutic functionality remain limited. Building upon these observations, this review examines nanobiochar surface chemistry, electron transfer behavior, and redox-active properties that may influence ROS regulation at biological interfaces. Particular emphasis is placed on biointerface interactions, including protein adsorption, protein corona formation, cellular uptake pathways, and the influence of hydrogel-mediated exposure on biological responses. The review further evaluates potential antimicrobial mechanisms, redox-sensitive signaling pathways, cytocompatibility assessment strategies, and the behavior of nanobiochar-containing systems under physiologically relevant conditions. Current evidence indicates a strong reliance on chemical antioxidant assays and short-term viability measurements, while mechanistic investigations involving intracellular ROS regulation, inflammatory signaling, mitochondrial function, and gene expression responses remain scarce. Collectively, the literature discussed in this article highlights the substantial gap between material development and biological validation and provides a framework for future studies aimed at evaluating the suitability of nanobiochar–hydrogel systems for wound-healing and regenerative applications.

## 1. Introduction

The growing demand for sustainable and multifunctional biomaterials has stimulated interest in carbon-based materials derived from renewable biomass. Among these, biochar has been widely utilized in environmental remediation, agriculture, and adsorption-based applications due to its porous structure, physicochemical stability, and tunable surface chemistry [1]. Recent advances in nanotechnology have enabled the production of nanobiochar, a nanoscale form of biochar produced from biomass through thermochemical conversion (typically pyrolysis), followed by physical, chemical, or mechanical processing to reduce the particle size to the nanometer range. Compared with conventional biochar, nanobiochar exhibits a substantially higher specific surface area, a greater density of oxygen-containing functional groups, improved colloidal stability, and enhanced surface reactivity. It can also be distinguished from engineered carbon nanomaterials such as graphene and carbon nanotubes because it is biomass-derived and retains a heterogeneous porous carbon architecture together with mineral constituents inherited from the parent feedstock. These properties contribute to its unique biological interactions and distinguish nanobiochar from conventional biochar and other engineered carbon nanomaterials.

These aspects have expanded interest in nanobiochar beyond environmental applications and prompted exploration of its potential use in antimicrobial materials, drug delivery systems, tissue engineering, and wound-healing platforms [2].

Hydrogels are among the most extensively investigated biomaterials for regenerative medicine because of their high water content, biocompatibility, and ability to mimic the extracellular matrix. Their three-dimensional architecture provides a hydrated microenvironment that supports cell growth, tissue regeneration, and localized therapeutic delivery [3]. Integrating nanobiochar within hydrogel matrices offers an opportunity to combine the structural advantages of hydrogels with the unique physicochemical and redox-active properties of nanobiochar. Such composite systems may provide enhanced antimicrobial activity, adsorption capacity, mechanical stability, and biological functionality. These characteristics are particularly attractive for wound-healing applications, where biomaterials must simultaneously maintain a moist environment, regulate oxidative stress, control microbial colonization, and support tissue repair [4].

A key property of nanobiochar is its potential role in redox regulation. The presence of graphitic domains, structural defects, and oxygen-containing functional groups enables its participation in electron transfer reactions and interactions with ROS [5]. Since ROS play critical roles in inflammation, antimicrobial defense, cellular signaling, and tissue regeneration, nanobiochar-mediated redox activity may significantly influence biological responses [6]. However, despite growing interest in nanobiochar-based biomaterials, the mechanisms governing these interactions remain poorly understood.

Current research is dominated by material synthesis, physicochemical characterization, adsorption performance, and basic antimicrobial evaluation. Biological assessments are frequently limited to chemical antioxidant assays and short-term viability measurements, providing little insight into intracellular ROS regulation, protein corona formation, cellular uptake, inflammatory responses, mitochondrial function, or redox-sensitive signaling pathways. Furthermore, the influence of hydrogel-mediated exposure and physiological conditions on nanobiochar behavior remains largely unexplored. This gap between material characterization and biological validation represents a major barrier to the rational development of nanobiochar-based therapeutic systems.

Therefore, the current review critically evaluates the potential of nanobiochar–hydrogel systems for therapeutic applications through bibliometric analysis and mechanistic assessment of the current literature. Particular emphasis is placed on redox modulation, biointerface interactions, antimicrobial activity, cellular signaling pathways, cytocompatibility, and behavior under physiological conditions. By identifying critical knowledge gaps and methodological limitations, the collective evidence aims to provide a framework for future studies investigating the suitability of nanobiochar–hydrogel systems for wound-healing and regenerative applications.

## 2. Bibliometric Landscape and Data-Driven Gap Identification

**Figure 1:**
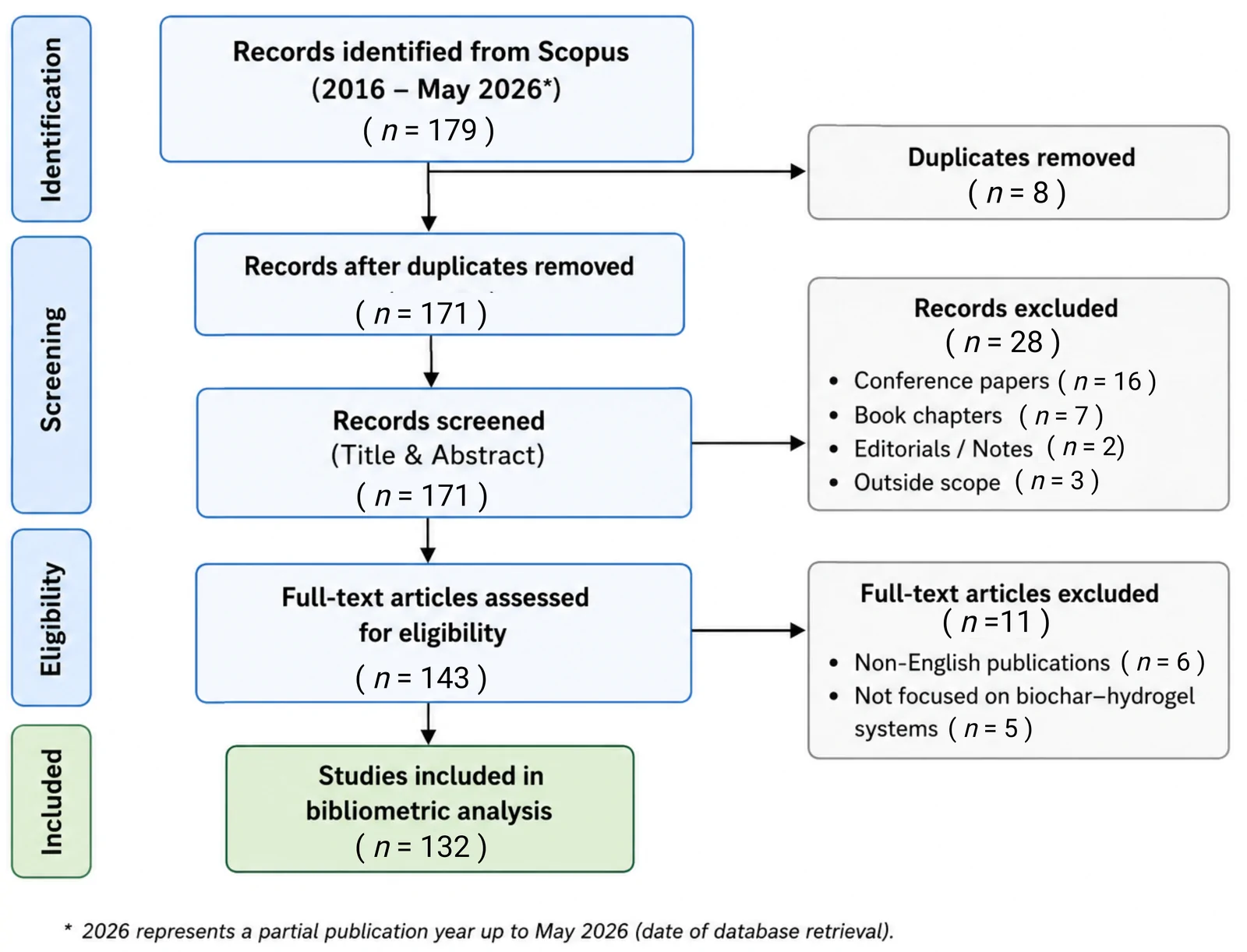
PRISMA-inspired flow diagram of the literature search and study selection process for the bibliometric analysis.

To evaluate the current research landscape and identify knowledge gaps relevant to nanobiochar-based therapeutic systems, a bibliometric analysis was conducted using the Scopus database. The search was performed using the keywords (“biochar” OR “nanobiochar” OR “nano-biochar”) AND (“hydrogel” OR “hydrogels”) within article titles, abstracts, and author keywords. The Scopus database was searched on 15 May 2026, covering publications from 2016 to May 2026, and the initial search retrieved 179 records. After removing 8 duplicate records, 171 unique records were retained for title and abstract screening. Following this screening, 28 records were excluded (16 conference papers, 7 book chapters, 2 editorials/notes, and 3 studies outside the review scope), leaving 143 articles for full-text eligibility assessment. After full-text evaluation, 11 articles were excluded (6 non-English publications and 5 not focused on nanobiochar–hydrogel systems). Consequently, 132 studies met the inclusion criteria and were retained for the bibliometric analysis. Bibliometric analyses, including publication trends, source analysis, country distribution, and keyword co-occurrence mapping, were performed using the Bibliometrix package and the Biblioshiny web interface implemented in RStudio (RStudio version 2026.07.1 (Build 147)) [7]. Because publications specifically addressing nanobiochar–hydrogel composites for biomedical applications remain limited, the broader biochar–hydrogel literature was analyzed to identify prevailing research directions, thematic gaps, and emerging opportunities relevant to the development of nanobiochar-based therapeutic systems. The literature selection process is summarized in the PRISMA-like flow diagram (Figure 1), which outlines the identification, screening, eligibility assessment, and final inclusion of studies used for the bibliometric analysis.

### 2.1. Publication Growth Trends and Research Expansion

**Figure 2:**
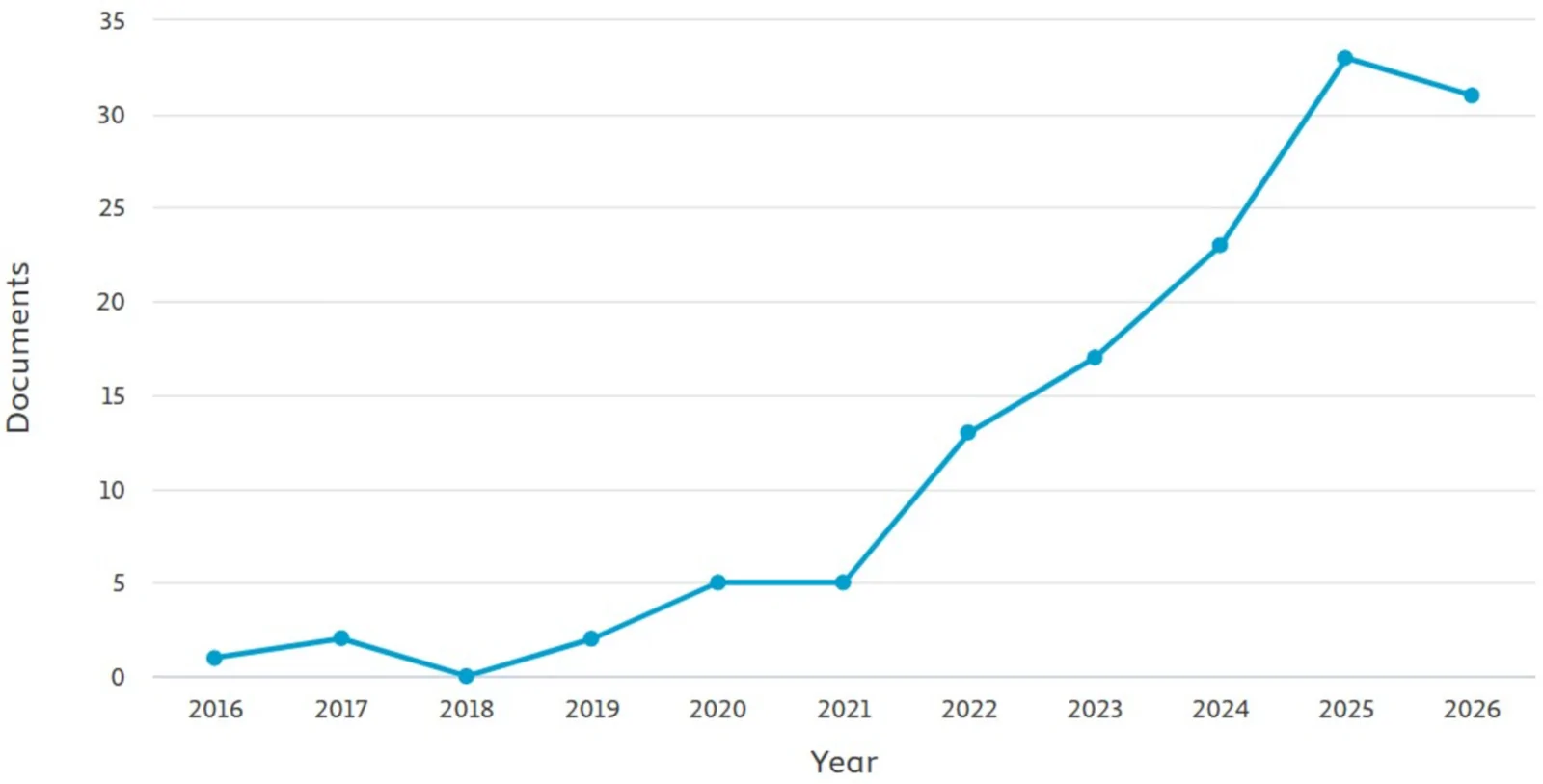
Annual scientific production of biochar–hydrogel research (2016–2026).

The annual publication trend shows a clear increase in research activity over the last decade (Figure 2). Between 2016 and 2021, publication output remained relatively low, with fewer than five articles published annually. A substantial increase was observed after 2021, reaching more than 30 publications per year by 2025. The 2026 data represent publications indexed up to May 2026 and therefore reflect a partial publication year. This growth reflects increasing scientific interest in biochar–hydrogel composites and their potential applications. However, the increase in publications has been driven primarily by environmental and engineering studies rather than biomedical investigations. Thus, while the field has expanded considerably, biological and therapeutic evaluations have not progressed at the same pace.

### 2.2. Subject Area Distribution and Research Focus

**Figure 3:**
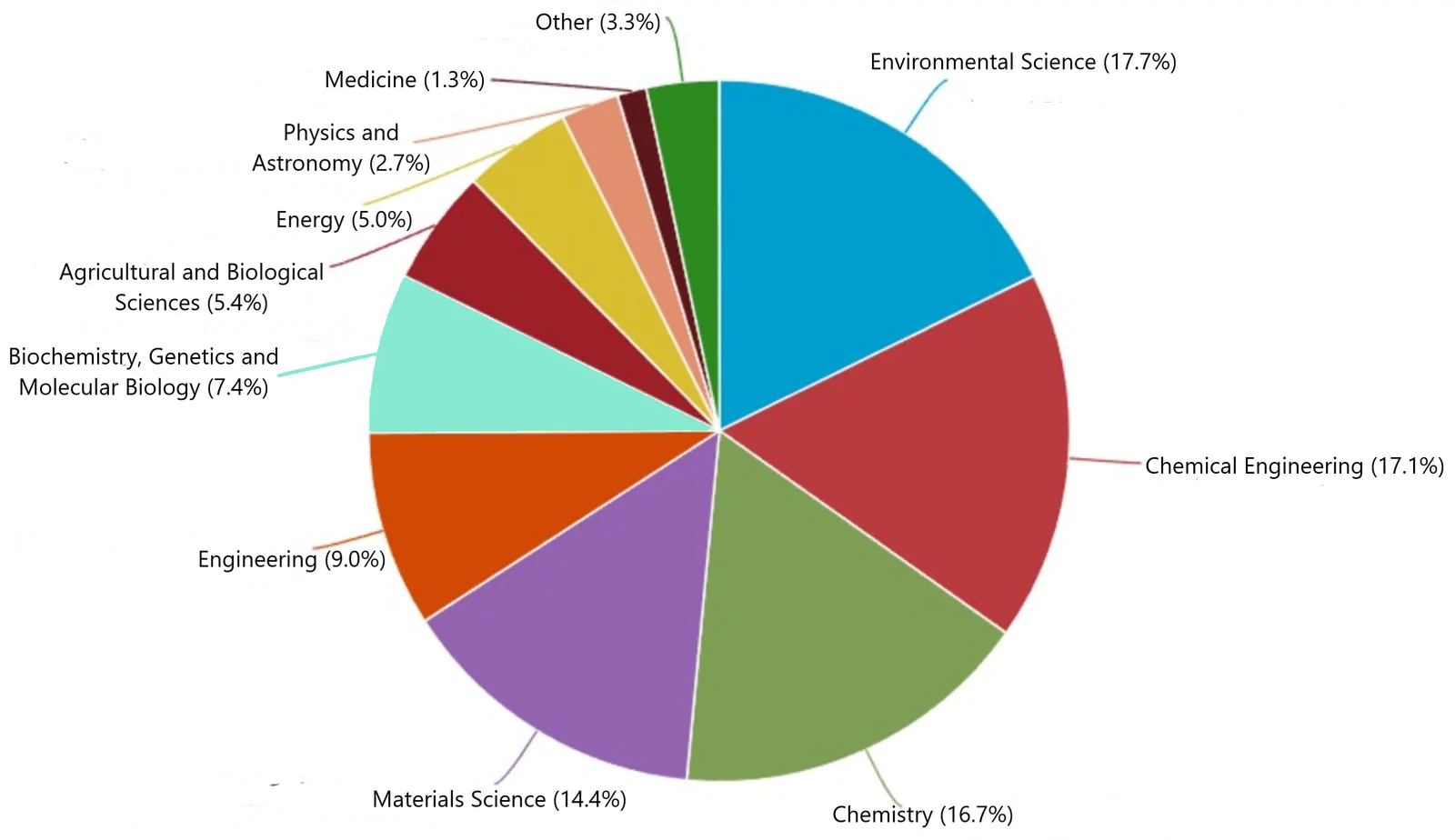
Subject area distribution of publications on biochar–hydrogel systems.

The subject area distribution highlights the current direction of research (Figure 3). Environmental Science (17.7%), Chemical Engineering (17.1%), Chemistry (16.7%), and Materials Science (14.4%) account for the majority of publications. In contrast, medicine-related studies represent only a small fraction of the literature. This distribution indicates that most investigations focus on material synthesis, physicochemical characterization, pollutant removal, and water treatment applications. Biological evaluation, therapeutic functionality, and cell–material interactions remain relatively underexplored. The limited contribution from medicine and life science disciplines suggests that translation toward biomedical applications is still in its infancy.

### 2.3. Keyword Co-Occurrence Analysis

The keyword co-occurrence network reveals several dominant research clusters. The largest cluster is centered on adsorption, indicating that pollutant removal remains the primary application of biochar–hydrogel composites. Other major clusters include water pollutants, wastewater treatment, chemistry, and material characterization techniques such as Fourier transform infrared spectroscopy and scanning electron microscopy (SEM).

**Figure 4:**
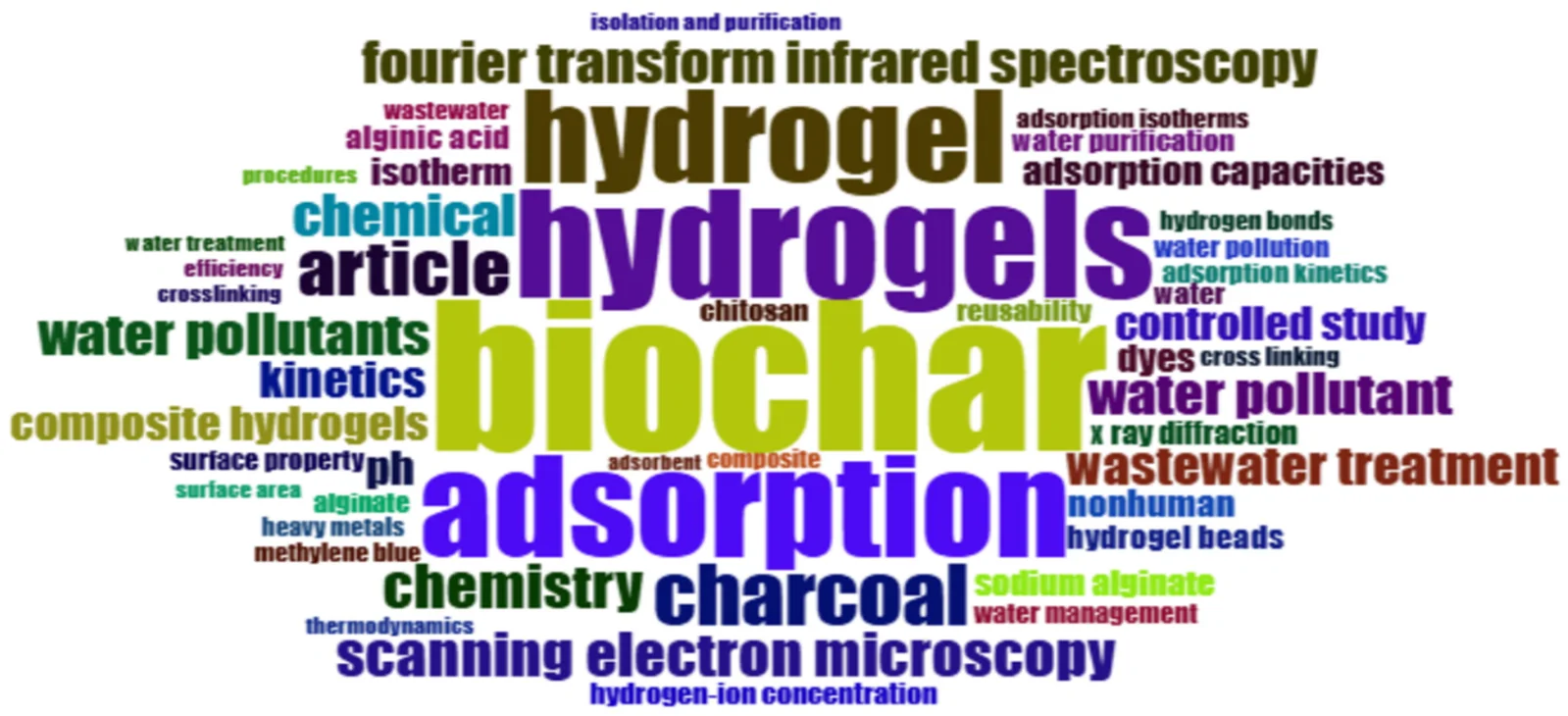
Word cloud of the most frequently occurring keywords in the retrieved literature.

**Figure 5:**
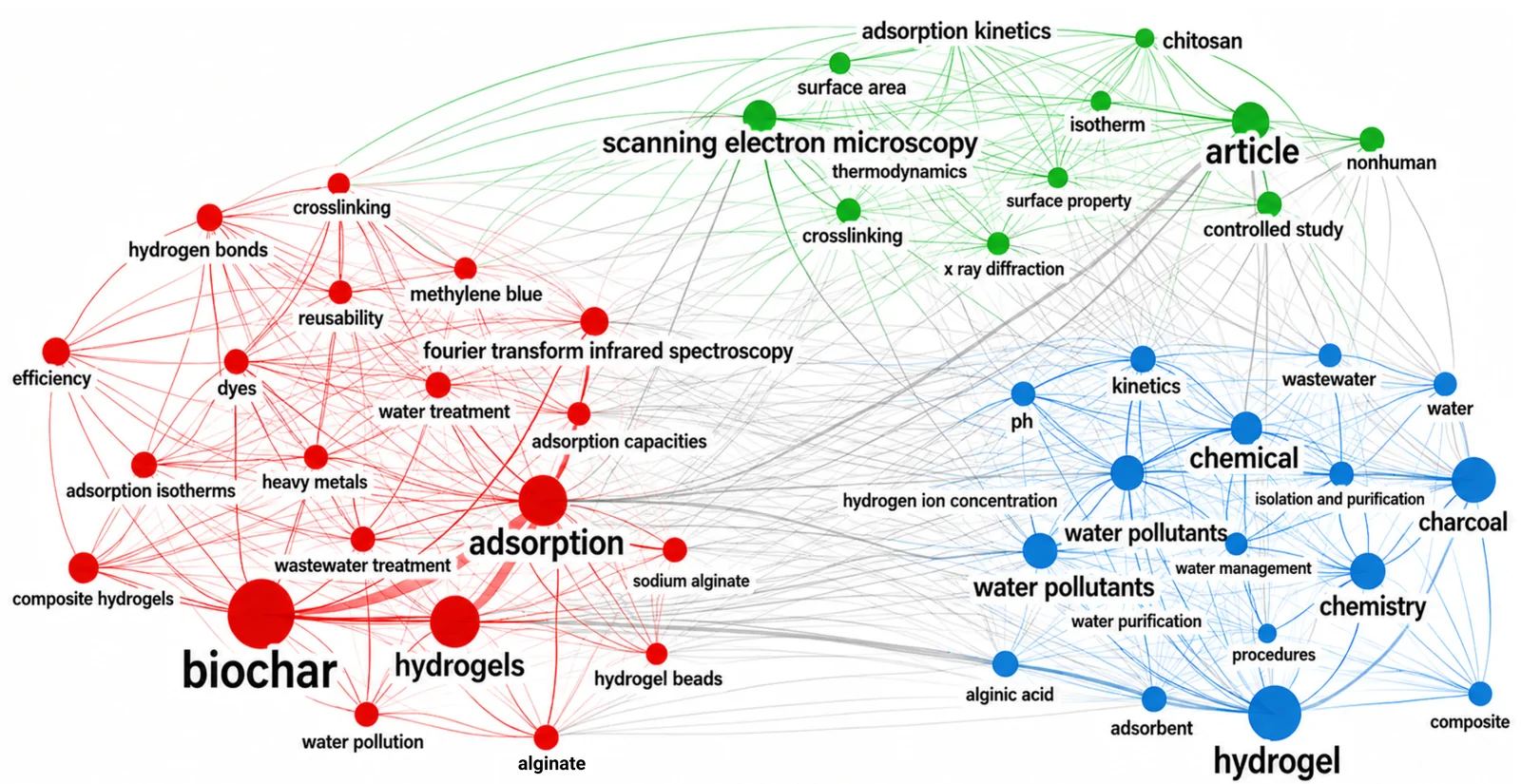
Keyword co-occurrence network showing major research themes in biochar–hydrogel research.

The word cloud analysis further supports these observations (Figure 4), where terms such as biochar, hydrogels, adsorption, water pollutants, wastewater treatment, and spectroscopy dominate the literature. Collectively, these findings demonstrate that the field is strongly oriented toward environmental remediation. Notably, keywords associated with biomedical applications are largely absent. Terms related to ROS, oxidative stress, inflammation, cytocompatibility, cellular uptake, signaling pathways, tissue engineering, and therapeutic delivery were not identified among the dominant or highly connected research themes. Likewise, keywords specifically related to nanobiochar are relatively uncommon despite growing interest in nanoscale biochar materials (Figure 5).

### 2.4. Geographic Distribution of Research Activity

**Figure 6:**
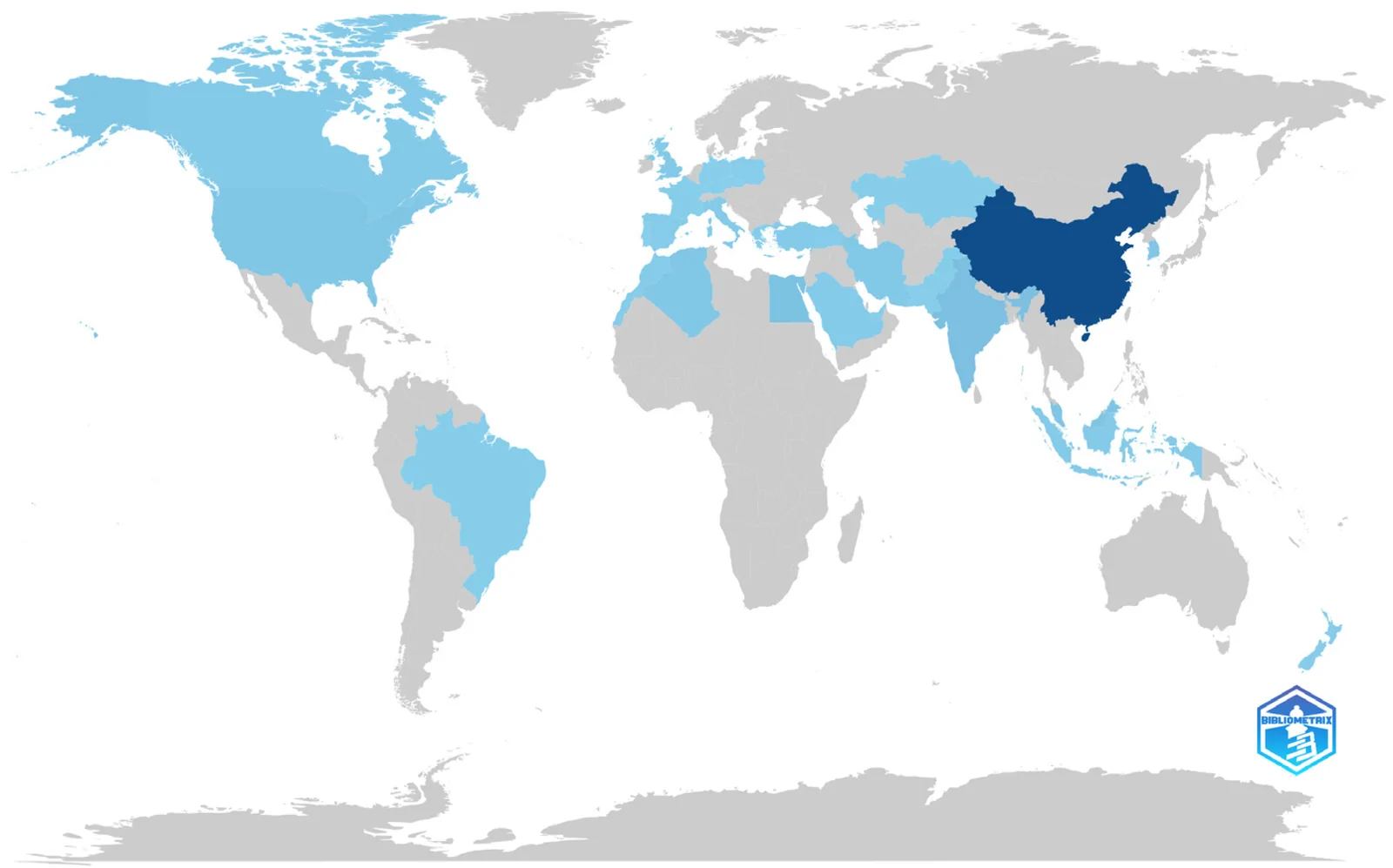
Global distribution of scientific production in biochar–hydrogel research. Darker blue indicates greater publication output; lighter blue indicates lower publication output; and gray denotes countries not represented in the dataset.

Country-level analysis revealed that China is the leading contributor to this research area, followed by the United States, India, and several European countries (Figure 6). The strong contribution from China reflects major investments in sustainable materials, biochar production, and environmental remediation technologies.

In contrast, contributions from the Middle East remain relatively limited compared with those from Asia, Europe, and North America. This observation reflects the current geographical distribution of research activity identified in the bibliometric analysis and highlights opportunities for broader international participation in the development of nanobiochar–hydrogel systems, particularly in emerging research regions.

### 2.5. Thematic Evolution

**Figure 7:**
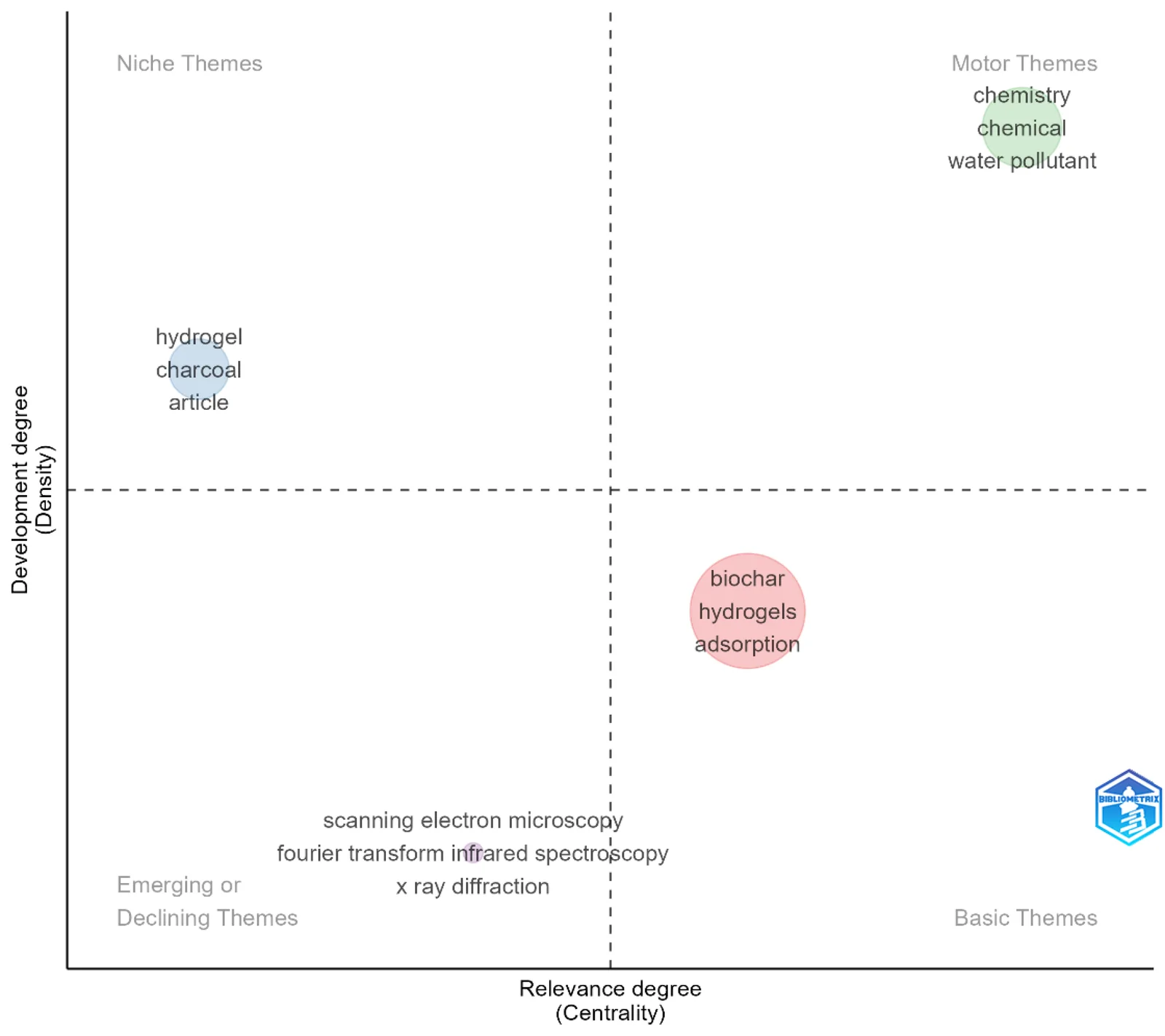
Thematic map showing the development and relevance of major research themes in biochar–hydrogel research.

The thematic map provides further insight into the maturity of research themes within the field (Figure 7). The cluster containing biochar, hydrogels, and adsorption occupies the basic theme region, indicating that these topics represent the established core of current research. In contrast, characterization-related themes, including Fourier transform infrared spectroscopy, X-ray diffraction, and SEM, appear as supporting or emerging themes associated with material development studies. Importantly, no major thematic clusters were identified around biological evaluation, redox biology, biointerface interactions, cellular signaling, or therapeutic applications. The absence of these themes suggests that the field remains largely focused on material performance rather than biological functionality.

### 2.6. Critical Knowledge Gaps

The bibliometric analysis reveals a clear imbalance between material development and biological evaluation in the current biochar–hydrogel literature. While considerable effort has been devoted to material synthesis, physicochemical characterization, and environmental applications, significantly less attention has been given to understanding the biological behavior of these systems.

Several important knowledge gaps emerge from the existing literature. First, the mechanisms underlying nanobiochar-mediated redox regulation remain poorly understood, with most studies relying on chemical antioxidant assays rather than biologically relevant intracellular assessments. Second, limited information is available regarding biointerface interactions, including protein adsorption, protein corona formation, cellular uptake, and intracellular fate. Third, the effects of nanobiochar on cellular signaling pathways involved in oxidative stress, inflammation, and cell survival have received little attention. Finally, current biological evaluations are often restricted to short-term cytotoxicity assays, with insufficient investigation of oxidative stress biomarkers, inflammatory responses, and long-term cytocompatibility. Another notable gap is the limited exploration of nanobiochar–hydrogel systems for therapeutic and regenerative applications. Despite the growing interest in hydrogels as biomedical platforms, the potential contribution of nanobiochar to redox modulation, antimicrobial activity, and biological functionality remains largely unexplored.

The bibliometric analysis further revealed the absence of studies directly investigating the in vitro biological or therapeutic evaluation of nanobiochar–hydrogel composite systems. While a limited number of studies have explored nanobiochar in biological contexts, these investigations remain fragmented and do not evaluate nanobiochar integrated within hydrogel matrices. Consequently, the following sections distinguish between mechanisms that are well demonstrated in related nanomaterial systems, those that are preliminarily suggested by the available nanobiochar literature, and those that remain largely unexplored in nanobiochar–hydrogel composites. Representative studies highlighting the current evidence base, biological evaluation, and remaining knowledge gaps in biochar/nanobiochar–hydrogel research are summarized in Table 1.

## 3. Surface Chemistry-Driven Redox Mechanisms

The therapeutic potential of nanobiochar–hydrogel systems is fundamentally governed by the surface chemistry of nanobiochar, which controls electron transfer reactions, reactive oxygen species (ROS) generation, and redox interactions at biological interfaces. Unlike conventional biomaterials that primarily provide structural support, nanobiochar possesses a heterogeneous carbon architecture composed of graphitic domains, structural defects, oxygen-containing functional groups, and residual mineral constituents. These features collectively determine the capacity of nanobiochar to participate in oxidation–reduction reactions through electron donation, electron acceptance, and electron-shuttling mechanisms [16].

The redox properties of nanobiochar are particularly important for biomedical applications because oxidative stress plays a central role in inflammation, tissue repair, microbial inactivation, and cellular homeostasis. Depending on its surface composition and environmental conditions, nanobiochar may either scavenge reactive oxygen species or facilitate their generation through catalytic and electron transfer processes [2]. However, despite increasing interest in the antioxidant properties of biochar-derived nanomaterials, most studies continue to rely on simplified chemical assays that provide limited insight into the underlying redox mechanisms. Therefore, understanding how specific surface characteristics govern electron transfer and ROS interactions is essential for the rational design of nanobiochar–hydrogel therapeutic systems [17]. When incorporated within hydrogel networks, these redox-active surface functionalities may experience altered diffusion dynamics, hydration states, and local oxygen availability. Such changes may influence electron transfer behavior and ROS interactions, potentially enabling more localized and sustained redox modulation compared with free nanobiochar particles [17,18].

### 3.1. Defect Sites and Electron Transfer Behavior

The redox behavior of nanobiochar originates from its unique carbon structure, which contains varying proportions of graphitic carbon, amorphous carbon, oxygen-containing functional groups, and structural defects generated during pyrolysis. These structural features act as active redox centers capable of participating in electron transfer reactions [10].

Among these components, oxygen-containing functional groups play a particularly important role. Quinone, hydroquinone, phenolic, carbonyl, hydroxyl, and carboxyl groups can undergo reversible oxidation–reduction reactions, enabling nanobiochar to function as an electron donor, electron acceptor, or electron shuttle. In addition to functioning as electron donors and acceptors, quinone–hydroquinone redox couples present on nanobiochar surfaces may facilitate electron shuttling between oxidized and reduced species, thereby sustaining continuous redox cycling and influencing ROS dynamics [19]. Li et al. (2026) demonstrated that these redox-active functionalities contribute significantly to the electron transfer capacity of biochar, facilitating redox reactions across diverse environmental systems [19]. Similarly, Baojun et al. (2025) reported that oxygen-containing functional groups promote reductive electron transfer processes by supporting catalytic iron regeneration and enhancing electron mobility [20]. Graphitic domains further contribute to redox activity by providing conductive pathways for long-range electron transport. Increased graphitization improves electrical conductivity and facilitates electron movement across the carbon framework, thereby enhancing electron transfer efficiency and redox activity [21]. These conductive regions support oxidation–reduction reactions without necessarily producing highly reactive radical intermediates.

Structural defects and edge sites are increasingly recognized as highly reactive regions within carbonaceous materials. Defective carbon atoms possess altered electronic distributions and unsaturated bonding configurations that create favorable sites for electron exchange [21]. Cheng et al. demonstrated that defect-rich biochar surfaces promote the formation of surface ROS during catalytic ozonation processes, highlighting the importance of defect engineering in regulating redox behavior [22]. Computational and electrochemical studies further indicate that defect sites preferentially adsorb oxygen molecules and electron acceptors, thereby facilitating redox reactions more effectively than ordered graphitic regions [23].

Despite growing evidence linking surface defects to electron transfer activity, their influence on biological performance remains poorly understood. Most available studies focus on environmental catalysis and pollutant degradation rather than biomedical applications. Consequently, direct relationships between defect density, electron transfer behavior, and therapeutic outcomes remain largely unexplored. Future investigations integrating electrochemical characterization with biological evaluation will be necessary to establish these connections.

### 3.2. Chemical Radical Scavenging Versus Intracellular Redox Modulation

Chemical assays such as 2,2-diphenyl-1-picrylhydrazyl (DPPH) and 2,2′-azino-bis (3-ethylbenzothiazoline-6-sulfonic acid) (ABTS) are commonly used to evaluate the antioxidant potential of nanobiochar, whereas analyses of electron transfer behavior, quinone/hydroquinone moieties, graphitic domains, and defect sites provide insight into its underlying redox-active surface chemistry [24,25]. However, these measurements should not be taken as direct evidence of intracellular antioxidant activity or therapeutic efficacy. As they are performed in simplified cell-free conditions, they generally reflect electron-donating and radical-scavenging activity toward synthetic radicals [24,25]. By contrast, biological redox regulation happens in a complex cellular environment in which ROS behave not just as damaging oxidants but also as signaling mediators in proliferation, differentiation, inflammation, and tissue repair [24,25]. Therefore, strong radical-scavenging activity in chemical assays alone cannot confirm that nanobiochar beneficially regulates intracellular ROS homeostasis [25,26].

A useful comparison can be drawn with polydopamine nanoparticles, whose redox-active catechol/quinone surface chemistry has been extensively investigated. Their biological performance depends not only on intrinsic antioxidant properties but also on particle size, surface chemistry, and interactions with the surrounding biological microenvironment [26,27,28]. These studies demonstrate that the biological activity of redox-active nanomaterials cannot be predicted solely from chemical antioxidant assays but requires complementary biological evaluation. Similar comprehensive investigations are still lacking for nanobiochar–hydrogel systems. Significantly, their biological effects have been connected to broader mechanisms, comprising intracellular ROS regulation, mitochondrial protection, modulation of antioxidant defense pathways, metal-ion chelation, and suppression of oxidative stress-related inflammation [26]. This example highlights that the biological performance of redox-active nanomaterials depends on dynamic cell–material interactions and microenvironmental conditions rather than on chemical antioxidant assays alone [26].

Accordingly, evaluation of nanobiochar–hydrogel systems should integrate physicochemical redox characterization with cell-based validation to establish whether biologically meaningful redox modulation occurs. Important assessments comprise intracellular and mitochondrial ROS measurements; activities of antioxidant enzymes such as superoxide dismutase (SOD), catalase (CAT), and glutathione peroxidase (GPx); oxidative stress biomarkers including glutathione (GSH), malondialdehyde (MDA), and lipid peroxidation products; and inflammatory mediators such as tumor necrosis factor-alpha (TNF-α), interleukin-6 (IL-6), and interleukin-1β. Furthermore, gene- and protein-expression analyses of redox-sensitive pathways, particularly Nrf2, NF-κB, and MAPK, using methods such as RT-qPCR, Western blotting, or RNA sequencing, are required to determine whether nanobiochar actually modulates intracellular redox homeostasis and supports therapeutic applications. Incorporating these biological readouts with conventional chemical antioxidant assays will provide a more rigorous framework for assessing nanobiochar-based biomaterials [25,26].

### 3.3. Surface Charge and Interaction Under Physiological Conditions

The redox behavior of nanobiochar is strongly influenced by surface charge and environmental conditions. Surface charge arises primarily from ionizable oxygen-containing functional groups whose protonation states vary with pH. Consequently, changes in pH can alter surface reactivity, colloidal stability, and electron transfer behavior [10,28]. Under physiological conditions, ionic strength and electrolyte composition further influence nanobiochar behavior. Dissolved ions can reduce electrostatic repulsion between particles, promoting aggregation and altering the available surface area for redox reactions [29]. Surface charge also affects the adsorption of oxygen molecules, oxidants, and biomolecules, thereby influencing electron transfer efficiency and ROS generation [30].

Once nanobiochar enters biological fluids, its physicochemical identity rapidly changes due to the adsorption of proteins and other biomolecules. These adsorbed layers may modify surface charge, mask reactive sites, and alter electron transfer processes [31,32]. Consequently, redox properties measured in simple aqueous systems may differ substantially from those exhibited under physiological conditions. Surface charge also influences interactions with cellular membranes and biological interfaces. Variations in charge density can affect adsorption, cellular association, and biological distribution, thereby indirectly influencing redox behavior [33]. However, studies investigating nanobiochar under physiologically relevant conditions remain limited. Most current evaluations are performed in simplified aqueous systems that fail to reproduce the complexity of biological environments.

**Figure 8:**
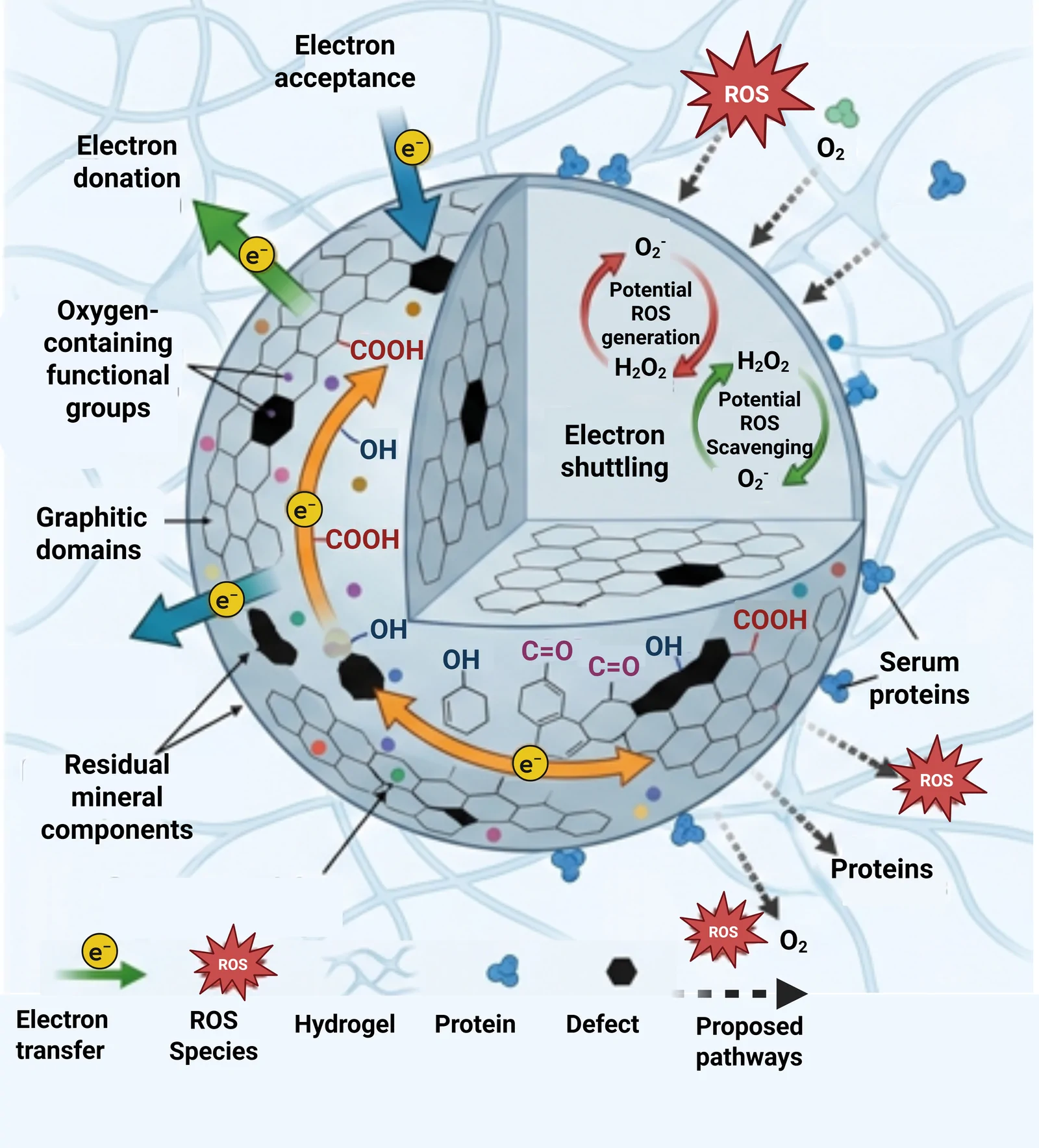
Conceptual schematic illustrating the surface chemistry-driven redox behavior of nanobiochar–hydrogel systems, including experimentally established surface characteristics and proposed redox pathways that may influence ROS generation and scavenging under physiological conditions.

Therefore, future investigations should assess nanobiochar redox behavior in physiologically relevant media containing serum proteins, biologically relevant ionic strengths, and dynamic exposure conditions. Such studies will be essential for understanding how surface chemistry translates into biological performance under realistic therapeutic conditions. The collective influence of graphitic domains, oxygen-containing functional groups, defect sites, and residual mineral components on electron transfer behavior is conceptually illustrated in Figure 8. The proposed redox pathways highlight potential mechanisms by which nanobiochar may modulate ROS under physiological conditions and require further biological validation.

## 4. Biointerface Interactions and Cellular Uptake

The biological performance of nanobiochar–hydrogel systems is ultimately determined by their interactions at the biointerface. Following exposure to physiological environments, nanobiochar encounters proteins, extracellular matrix components, cellular membranes, and biological fluids that collectively influence its biological identity and subsequent cellular responses. These interactions govern adsorption, cellular recognition, internalization, intracellular trafficking, and biodistribution, ultimately affecting cytocompatibility, redox activity, and therapeutic efficacy. Unlike physicochemical characterization performed in simplified aqueous systems, biointerface interactions are highly dynamic and continuously evolve in response to the surrounding biological environment [34]. Consequently, understanding these processes is essential for predicting the biological behavior of nanobiochar-based therapeutic systems.

### 4.1. Interaction with Cell Membranes

Cell membranes constitute the first biological barrier encountered by nanobiochar following exposure to living systems. Initial interactions are governed by electrostatic forces, hydrophobic interactions, hydrogen bonding, and van der Waals attractions between the nanoparticle surface and membrane components. The magnitude of these interactions is strongly influenced by particle size, surface charge, surface chemistry, and hydrophobicity [33,35].

Adsorption onto cell membranes represents the first step in the cellular response to nanobiochar exposure. The physicochemical characteristics of the particle surface influence the extent of membrane association and determine whether particles remain attached to the membrane surface or undergo subsequent internalization. Nanomaterials possessing favorable surface characteristics may induce membrane wrapping, receptor engagement, and endocytic uptake, whereas unfavorable interactions can reduce cellular association [33,34].

These interactions are further influenced by the dynamic biological environment. Most experimental studies evaluating particle–cell interactions are performed under serum-free or simplified conditions, which may not accurately represent physiological environments. In biological fluids, nanoparticles rapidly adsorb proteins and other biomolecules to form a biomolecular (protein) corona, which alters surface properties and modifies the interaction profile between nanoparticles and cellular membranes [31,32]. Consequently, observations obtained under simplified experimental conditions may not accurately predict biological responses in vivo.

To date, direct investigations of nanobiochar interactions with mammalian cell membranes remain extremely limited. The available biochar-related evidence is largely restricted to environmental studies, such as the interaction of biochar with *Chlorella vulgaris* in the presence of nanoplastics or silver nanoparticles, which demonstrate that biochar surface properties can influence biological interactions but do not evaluate membrane interactions in mammalian or therapeutic systems [35]. Consequently, the membrane interaction mechanisms discussed above are primarily inferred from studies on engineered nanoparticles and related carbon nanomaterials [33,34] and represent biologically plausible pathways that require direct experimental validation in nanobiochar–hydrogel systems.

### 4.2. Protein Corona Formation

Immediately after entering biological fluids, engineered nanoparticles are rapidly coated with proteins, lipids, and other biomolecules, forming a dynamic interfacial layer commonly referred to as the protein corona. This adsorbed layer effectively defines the biological identity of the material and determines how cells recognize and respond to the nanoparticle [36].

Protein coronas are generally classified into hard and soft corona layers. The hard corona consists of strongly bound proteins that remain associated with the nanoparticle surface for extended periods, whereas the soft corona contains loosely bound proteins that continuously exchange with surrounding biomolecules. The composition and stability of these layers depend on surface chemistry, charge, roughness, hydrophobicity, particle size, and the composition of the biological environment [37,38].

The biological consequences of protein corona formation are substantial. Adsorbed proteins can mask reactive surface functionalities, alter surface charge, modify colloidal stability, and influence cellular recognition pathways [37,39]. Depending on its composition, the corona may either facilitate receptor-mediated cellular uptake or reduce interactions through steric shielding effects [39,40]. Furthermore, the protein corona continues to evolve following cellular internalization, influencing intracellular trafficking and biological activity [31].

Nanobiochar possesses a chemically heterogeneous surface containing graphitic domains, oxygen-containing functional groups, and defect-rich regions that provide numerous binding sites for biomolecules [23]. Consequently, substantial variability in protein corona composition can be expected depending on the biomass source, production conditions, and surface modification strategies [36]. However, to date, protein corona formation has not been systematically characterized for nanobiochar under physiological conditions. Therefore, the discussion presented above is primarily inferred from studies on engineered nanoparticles and related carbon nanomaterials [31,36,37,38,39,40]. Experimental characterization of the nanobiochar protein corona remains an important research priority for understanding biointerface interactions and subsequent biological responses.

### 4.3. Cellular Internalization Pathways

Following membrane association, engineered nanoparticles may enter cells through multiple endocytic pathways. For nanobiochar, however, these internalization mechanisms remain largely hypothetical and are inferred from the broader nanoparticle literature. Cellular internalization is influenced by particle size, morphology, surface chemistry, charge, and protein corona composition. The dominant uptake mechanism often depends on the physicochemical characteristics of the nanoparticle and the type of cell being investigated [41,42]. The most common uptake pathways include clathrin-mediated endocytosis, caveolin-mediated endocytosis, macropinocytosis, and phagocytosis. Rather than operating independently, these pathways frequently function simultaneously, with several uptake mechanisms contributing to nanoparticle internalization under a given set of conditions. Consequently, identifying a single dominant pathway is often challenging [41,42,43].

Many studies investigating nanoparticle uptake rely on pharmacological inhibitors to identify endocytic mechanisms. However, a recent study highlighted that such inhibitors frequently exhibit non-specific effects and may influence multiple cellular processes simultaneously, potentially leading to misleading conclusions. Consequently, complementary approaches, including live-cell imaging, fluorescent tracking, electron microscopy, and genetic manipulation techniques are increasingly recommended for accurately elucidating nanoparticle internalization pathways and intracellular trafficking [44].

Following internalization, nanoparticles may localize within endosomes, lysosomes, cytoplasmic compartments, or other intracellular structures. Accumulation within lysosomal compartments is particularly important because acidic conditions may alter nanoparticle surface chemistry and redox activity. Lysosomal processing can subsequently influence mitochondrial function and intracellular ROS generation, thereby affecting oxidative stress responses and downstream cellular signaling. However, whether similar intracellular trafficking events occur with nanobiochar remains largely unknown. To date, no studies have systematically characterized the endocytic uptake pathways, intracellular trafficking, or subcellular localization of nanobiochar in mammalian cells. Consequently, the uptake pathways and intracellular fate described above should be regarded as biologically plausible mechanisms inferred from studies on engineered nanoparticles rather than experimentally demonstrated responses of nanobiochar–hydrogel systems. Intracellular trafficking influences the duration of cellular exposure, degradation behavior, and interactions with biological machinery [41,44]. Consequently, understanding the intracellular fate of nanobiochar is essential for predicting its biological effects.

### 4.4. Influence of the Hydrogel Matrix on Biointerface Behavior

The hydrogel component of nanobiochar–hydrogel systems functions not only as a structural carrier but also as an active regulator of nanobiochar bioavailability and biointerface interactions. Hydrogels provide a hydrated three-dimensional environment that can regulate particle mobility, diffusion behavior, cellular exposure, and biomolecular interactions. Properties such as porosity, stiffness, mesh size (porosity), degradation rate, swelling behavior, surface charge, hydration, and surface topography actively regulate nanobiochar bioavailability and biological responses. For example, hydrogel stiffness influences cell adhesion, spreading, and mechanotransduction, whereas mesh size and swelling behavior determine nanoparticle diffusion, retention, and release kinetics. Similarly, degradation rate governs the duration of nanobiochar exposure, while surface charge and hydration affect protein adsorption, protein corona formation, and electrostatic interactions at the biointerface. Surface topography further modulates focal adhesion formation, cytoskeletal organization, and subsequent cell–material interactions [45,46,47].

Incorporation of nanobiochar within hydrogel matrices may reduce direct particle–cell contact while enabling controlled and localized exposure. This can potentially minimize acute cytotoxic effects while preserving beneficial biological functions. Hydrogels can also improve retention at target sites, prolong therapeutic activity, and reduce unintended systemic distribution [45].

The hydrogel network further influences protein adsorption and mass transport processes. By regulating the diffusion of proteins, nutrients, signaling molecules, and therapeutic agents, hydrogels create a dynamic microenvironment that can modify how cells interact with embedded nanobiochar particles. Surface patterning, mechanical tuning, and composite design strategies have been shown to influence cell adhesion, migration, proliferation, and differentiation in hydrogel-based systems [46,48]. Similarly, studies on polarized biomaterial surfaces have demonstrated that interfacial physicochemical properties can significantly regulate cell adhesion, further emphasizing the importance of biointerface engineering in controlling cell–material interactions [49].

Hemocompatibility and immune interactions represent additional components of the biointerface that should be considered when evaluating nanobiochar–hydrogel systems for biomedical applications. For wound dressings and implantable biomaterials, initial contact with blood and immune cells influences subsequent inflammatory responses, tissue integration, and overall biological safety. Material properties such as surface chemistry, charge, hydrophilicity, and protein adsorption can influence these interactions and should therefore be considered during the biological evaluation of biochar-based hydrogel systems [50,51]. However, direct investigations of hemocompatibility, complement activation, macrophage responses, or cytokine production have not yet been reported for nanobiochar–hydrogel systems. Consequently, these aspects remain important knowledge gaps and should be incorporated into future in vitro evaluation frameworks to establish the biological safety of these materials.

Recent studies have also demonstrated that biochar-modified hydrogels can facilitate interfacial electron transfer processes while simultaneously providing structural support and enhanced biological compatibility. Such multifunctional behavior highlights the potential of nanobiochar–hydrogel systems as active candidate therapeutic platforms rather than passive scaffolds [12].

Despite these promising developments, the influence of hydrogel confinement on protein corona formation, cellular uptake, intracellular trafficking, and long-term biological responses remains poorly understood.

**Figure 9:**
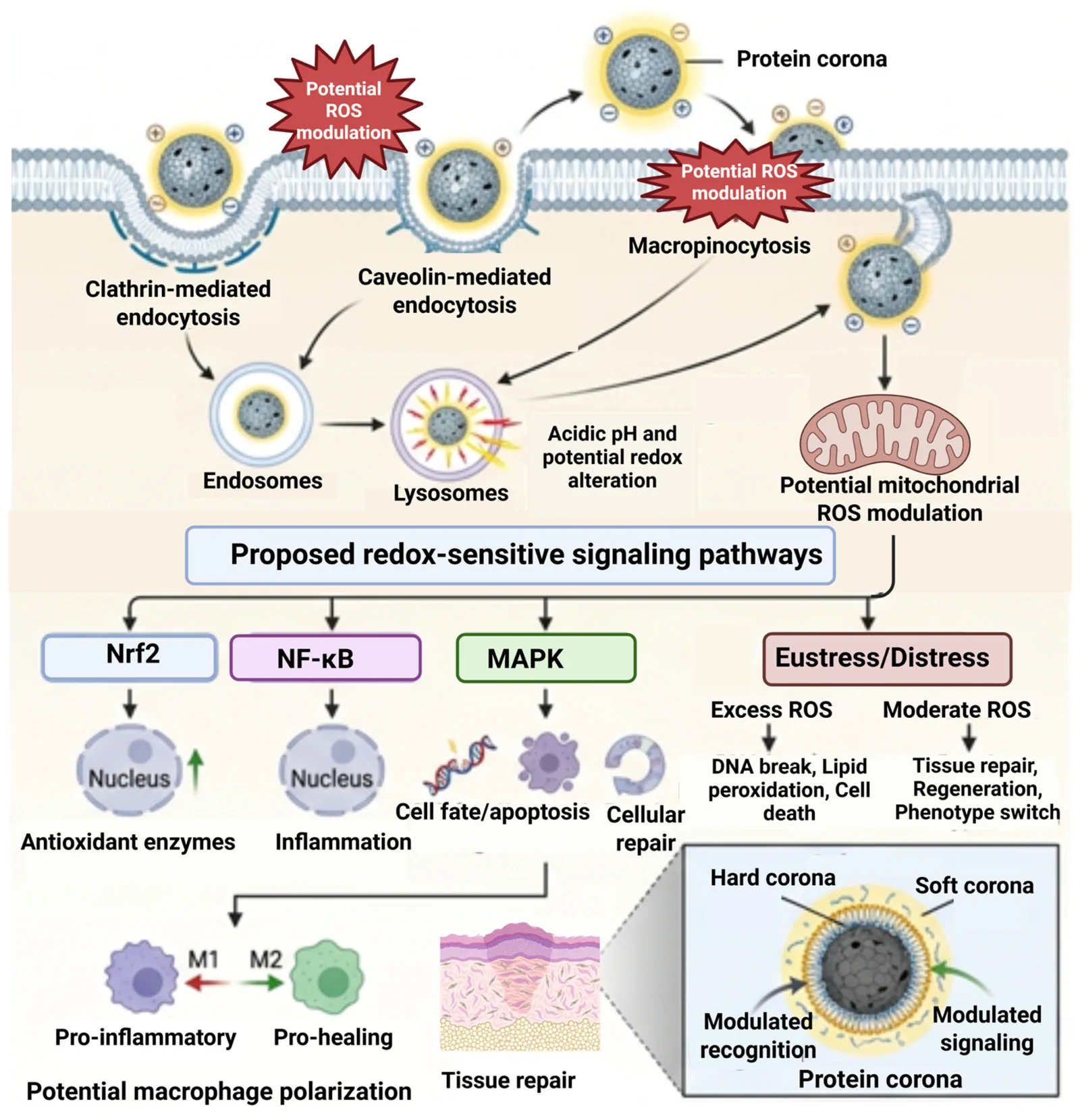
Conceptual schematic illustrating biointerface interactions and proposed downstream redox-sensitive signaling pathways associated with nanobiochar–hydrogel systems.

Collectively, biointerface interactions are expected to influence how nanobiochar is recognized, internalized, and processed by biological systems, although direct experimental evidence for nanobiochar–hydrogel systems remains limited. These events influence subsequent antimicrobial activity, cytocompatibility, redox regulation, and cellular signaling pathways. Therefore, a mechanistic understanding of biointerface behavior is essential for interpreting the biological effects of nanobiochar–hydrogel therapeutic systems and for guiding the development of more effective and predictable biomedical materials. The conceptual model presented in Figure 9 summarizes the interplay among protein corona formation, cellular internalization, intracellular trafficking, and ROS regulation in nanobiochar–hydrogel systems. It integrates experimentally established nanoparticle–cell interactions with proposed downstream biological pathways that require direct validation in nanobiochar–hydrogel systems.

## 5. Antimicrobial Mechanisms: Redox Interference and Cellular Damage

The antimicrobial activity of nanobiochar-based materials has attracted increasing attention due to their potential application in wound healing, infection control, tissue engineering, and drug delivery systems. Unlike conventional antimicrobial agents that often rely on a single mode of action, nanobiochar may exert antimicrobial effects through multiple interconnected mechanisms involving membrane disruption, oxidative stress, and interference with microbial electron transfer processes [52]. While nanobiochar–hydrogel composites have shown potential for biomedical and antimicrobial applications, the influence of hydrogel incorporation on antimicrobial mechanisms remains poorly characterized. Despite growing evidence of antimicrobial activity, the mechanistic basis of microbial inhibition remains insufficiently understood, and many studies continue to rely primarily on growth inhibition assays rather than detailed evaluations of membrane damage, oxidative stress, intracellular responses, and microbial metabolic alterations.

### 5.1. Membrane Disruption

Disruption of microbial membrane integrity is one of the most frequently proposed mechanisms underlying the antimicrobial activity of nanobiochar-based systems. Bacterial cell surfaces generally possess a net negative charge due to the presence of phospholipids, lipopolysaccharides, and teichoic acids. These surface characteristics facilitate interactions with nanobiochar through electrostatic attraction, hydrophobic interactions, and van der Waals forces [52]. Upon contact with microbial cells, nanobiochar particles may adsorb onto the outer membrane, creating localized stress at the cell surface [53].

The irregular morphology, rough surface texture, and sharp edges associated with nanoscale carbonaceous materials can physically damage microbial membranes, leading to increased permeability and leakage of intracellular contents. Such disruption compromises membrane integrity, alters ion transport, and interferes with essential physiological functions required for microbial survival. In some cases, membrane damage may be further amplified by oxidative processes occurring at the material–cell interface. ROS generated near the nanobiochar surface can oxidize membrane lipids and proteins, resulting in additional destabilization of the microbial envelope. Consequently, membrane disruption often represents the initial stage of a multifactorial antimicrobial response involving both physical and chemical mechanisms [54].

### 5.2. ROS-Mediated Oxidative Stress

ROS-mediated oxidative stress is another major mechanism proposed for nanobiochar-associated antimicrobial activity. Nanobiochar surfaces contain redox-active functional groups capable of participating in electron transfer reactions that generate ROS, including superoxide radicals (O_2_•^−^), hydroxyl radicals (•OH), and hydrogen peroxide (H_2_O_2_) [52]. While microorganisms possess antioxidant defense systems to regulate endogenous ROS levels, excessive ROS accumulation can overwhelm these protective mechanisms and trigger oxidative damage [55].

Elevated ROS levels can affect multiple cellular targets simultaneously. Lipid peroxidation compromises membrane fluidity and permeability, protein oxidation disrupts enzymatic activity and metabolic function, and DNA oxidation contributes to genomic instability and impaired replication [56]. Collectively, these effects interfere with microbial homeostasis and ultimately lead to cell death. ROS-mediated antimicrobial activity has been frequently proposed as an important mechanism underlying the biological effects of carbon-based nanomaterials and biochar-derived nanocomposites, particularly when combined with catalytic metal nanoparticles that enhance redox cycling reactions [57]. However, despite the frequent attribution of antimicrobial activity to oxidative stress mechanisms, direct quantification of ROS generation remains relatively uncommon in nanobiochar studies, limiting mechanistic understanding.

### 5.3. Electron Transfer Interference with Microbial Metabolism

In addition to causing direct oxidative damage, nanobiochar may influence microbial survival through interference with cellular electron transfer processes. Biochar-derived materials contain quinone–hydroquinone moieties, phenolic groups, persistent free radicals, and graphitic domains that exhibit electron transfer capabilities. These surface features enable nanobiochar to function as an electron shuttle capable of accepting, storing, and donating electrons during biological reactions [58].

Microbial respiration relies on tightly regulated electron transport chains that drive ATP synthesis and cellular energy production. Disturbance of electron flow can impair respiration, reduce ATP generation, and disrupt metabolic activity [59]. Emerging evidence from environmental microbiology suggests that biochar-mediated electron transfer can alter microbial redox balance and modulate energy metabolism by influencing electron transport processes and microbial respiratory activity [58]. In pathogenic microorganisms, disruption or diversion of electron transfer pathways may impair respiration, reduce ATP production, and contribute to antimicrobial effects [60].

Despite increasing recognition of these phenomena, the role of electron transfer interference in nanobiochar-mediated antimicrobial activity remains poorly characterized. Most current evidence originates from environmental and wastewater-related systems rather than biomedical applications. Consequently, further investigation is required to determine how nanobiochar influences microbial respiration, energy metabolism, and redox homeostasis under physiologically relevant conditions.

### 5.4. Synergistic Effects of Nanobiochar–Hydrogel Systems

The incorporation of nanobiochar into hydrogel matrices can substantially enhance antimicrobial performance through synergistic interactions between the carbonaceous nanomaterial and the surrounding polymer network [2]. Hydrogels are highly hydrated three-dimensional structures capable of maintaining prolonged contact between antimicrobial agents and microbial cells. This extended residence time can increase local exposure and improve antimicrobial efficacy [61].

The porous architecture of hydrogels facilitates the diffusion of oxygen, nutrients, and bioactive molecules while simultaneously enabling the sustained release of incorporated antimicrobial components. In nanobiochar–hydrogel systems, the hydrogel matrix functions not only as a carrier but also as a regulator of the local microenvironment surrounding infected tissues [62]. This can be particularly advantageous in wound-healing applications, where prolonged antimicrobial activity and controlled therapeutic delivery are desirable.

Recent advances have further enabled the development of stimuli-responsive hydrogels capable of responding to environmental triggers such as pH, temperature, or ROS levels. Such systems offer opportunities for controlled and site-specific antimicrobial activity. Moreover, combining nanobiochar-containing hydrogels with metal nanoparticles, antibiotics, antimicrobial peptides, or redox-active compounds can generate synergistic effects that exceed the performance of the individual components alone [63]. These multifunctional platforms therefore represent promising candidates for next-generation antimicrobial biomaterials.

### 5.5. Limitations of Current Antimicrobial Evaluation Approaches

Despite numerous reports describing the antimicrobial efficacy of nanobiochar-based materials, significant methodological limitations remain. Most studies continue to rely heavily on zone-of-inhibition (ZOI) assays as the primary measure of antimicrobial performance. Although these assays are simple, rapid, and inexpensive, they provide only limited mechanistic information. For nanobiochar materials, interpretation can be particularly challenging because particle diffusion through agar matrices may be restricted. Consequently, inhibition zone size may reflect diffusion characteristics rather than true antimicrobial potency. Furthermore, ZOI assays cannot distinguish between bacteriostatic and bactericidal effects and provide little information regarding the underlying mechanisms of microbial inhibition [64,65].

To address these limitations, future studies should incorporate more mechanistically informative approaches. Membrane integrity assays using fluorescent probes such as propidium iodide, SYTOX Green, and Live/Dead staining can provide direct evidence of membrane damage. Complementary imaging techniques, including SEM, transmission electron microscopy, and atomic force microscopy (AFM), enable visualization of structural alterations in microbial cell envelopes. Measurement of intracellular leakage products, including proteins, nucleic acids, and potassium ions, can further confirm membrane disruption and permeability changes [66].

Direct quantification of ROS is equally important for validating oxidative stress-mediated antimicrobial mechanisms. Fluorescent probes such as DCFH-DA, together with electron paramagnetic resonance techniques and oxidative stress biomarkers, can provide valuable information regarding radical generation and intracellular ROS accumulation. However, many studies continue to attribute antimicrobial activity to ROS generation without directly measuring ROS levels, making mechanistic interpretation difficult [67].

Another major limitation is the widespread use of planktonic bacterial cultures as experimental models. In clinical settings, many persistent infections are associated with biofilms rather than free-floating microorganisms. Biofilms are protected by extracellular polymeric matrices that limit antimicrobial penetration and enhance microbial tolerance to stress. As a result, antimicrobial activity observed against planktonic cells may not accurately predict efficacy against established biofilms [68]. Future investigations should therefore evaluate nanobiochar–hydrogel systems using biofilm formation, maturation, and disruption models that more closely mimic clinically relevant conditions.

Collectively, these observations indicate that current antimicrobial evaluations often provide evidence of efficacy but limited understanding of mechanism. A transition from simple growth inhibition assays toward integrated assessments incorporating membrane integrity analysis, ROS quantification, electron transfer measurements, and biofilm models will be essential for establishing a mechanistic understanding of nanobiochar-mediated antimicrobial activity. Such knowledge will facilitate the rational design of nanobiochar–hydrogel therapeutic systems with improved efficacy and clinical relevance.

### 5.6. Biofilm-Specific Evaluation: Moving Beyond Planktonic Activity

Biofilms are a major cause of chronic wound infection and impaired healing, with approximately 60–80% of chronic wounds containing biofilms [69] and a reported prevalence of 78.2% in meta-analysis studies [70]. Biofilm-associated bacteria exhibit much greater tolerance to antibiotics and immune responses than planktonic cells; therefore, nanobiochar–hydrogel antimicrobial systems require biofilm-specific evaluation rather than solely planktonic inhibition testing.

Crystal violet staining is widely used for biofilm biomass quantification [71,72], but it is insufficient alone for complex multi-species biofilms and should be combined with complementary methods [73]. Confocal laser scanning microscopy with live/dead staining (SYTO9/propidium iodide) enables three-dimensional visualization of biofilm structure, viability, thickness, and coverage. However, the performance of SYTO9/PI requires optimization because dye interactions and staining kinetics may influence the accuracy of viability measurements [74]. To complement staining-based approaches, label-free techniques, including optical coherence tomography (OCT), Raman spectroscopy, quartz crystal microbalance (QCM), and impedance-based sensing, provide real-time, non-destructive assessment of biofilm formation, structural development, and material–biofilm interactions. New metabolic probes, such as CAM/TMA 3,4-DPH, have shown strong correlations with colony-forming unit counts, indicating potential as alternatives [75].

Tetrazolium-based assays such as XTT are commonly used to measure biofilm metabolic activity. However, carbon nanomaterials require careful evaluation because functionalization of multi-walled carbon nanotubes (MWCNTs) can modify their antibiofilm or probiofilm effects. Studies using XTT have shown that pristine MWCNTs may not exhibit cytotoxicity toward biofilm-associated bacterial cells, underscoring the importance of biofilm-specific testing [76].

Microfluidic and lab-on-chip platforms allow biofilm growth under physiologically relevant shear stress and nutrient gradients, better representing wound conditions [77]. Chronic wound-mimicking models incorporating simulated wound fluid, collagen-based 3D matrices, and multi-species communities provide more realistic evaluation systems [78].

Biofilm inhibition studies evaluate the prevention of biofilm formation, whereas eradication studies assess the removal of established, mature biofilms [79]. Since many studies focus only on inhibition, their clinical relevance against chronic biofilms may be limited. Recent studies have shown that nanostructured lipid carriers can disrupt preformed *H. pylori* biofilms [80], and other compounds demonstrate concentration-dependent biofilm inhibition against enteroaggregative *E. coli* [81].

In nanobiochar–hydrogel systems, biofilm evaluation must account for the composite’s unique properties. The hydrogel may affect biofilm development through confinement, nutrient restriction, and altered transport, while nanobiochar may contribute to antibiofilm activity through proposed redox-mediated mechanisms that require experimental validation. Therefore, direct-contact assays using hydrogel-embedded nanobiochar are recommended, as extract-based methods may underestimate antibiofilm activity by failing to capture material–biofilm interactions at the hydrogel surface. The hydrogel’s ability to maintain local concentrations of redox-active species should also be considered when interpreting antibiofilm performance.

## 6. Cellular Signaling Pathways and Redox Biology

The biological effects of nanobiochar extend beyond direct interactions with cellular membranes and extracellular environments. Owing to its redox-active surface chemistry, nanobiochar has the potential to influence intracellular signaling pathways that regulate oxidative stress responses, inflammation, proliferation, apoptosis, and tissue repair [82]. These signaling pathways are highly sensitive to changes in ROS levels and collectively determine whether cells adapt to environmental stress or undergo dysfunction and death [83]. Although ROS generation and scavenging are frequently cited as important mechanisms underlying the biological activity of nanobiochar, the molecular pathways connecting these redox processes to cellular responses remain poorly understood. Consequently, understanding the relationship between nanobiochar-mediated redox modulation and intracellular signaling represents a critical step toward the development of biologically validated therapeutic systems. As illustrated in the conceptual model presented in Figure 9, intracellular ROS generated following nanobiochar internalization may potentially influence multiple redox-sensitive signaling pathways, including Nrf2, NF-κB, and MAPK networks. These pathways are inferred from the current evidence and require direct experimental validation in nanobiochar–hydrogel systems.

### 6.1. ROS as Regulators of Cellular Signaling

ROS are traditionally viewed as harmful by-products of cellular metabolism; however, extensive research has demonstrated that they also function as important signaling molecules. Under physiological conditions, low to moderate ROS concentrations regulate numerous biological processes, including cell proliferation, differentiation, migration, angiogenesis, and immune responses. These signaling functions are achieved through the reversible oxidation of proteins, enzymes, transcription factors, and signaling molecules that collectively coordinate cellular adaptation to environmental stimuli [84].

The biological outcome of ROS signaling is highly dependent on the concentration and duration of exposure. Controlled ROS generation can activate protective and regenerative pathways, whereas excessive ROS accumulation can overwhelm antioxidant defenses and induce oxidative stress. Such oxidative imbalance can lead to lipid peroxidation, protein oxidation, DNA damage, mitochondrial dysfunction, and activation of inflammatory signaling cascades [85].

Nanobiochar contains redox-active functional groups, graphitic domains, persistent free radicals, and quinone–hydroquinone structures capable of participating in electron transfer reactions. These physicochemical characteristics suggest that nanobiochar may influence intracellular ROS homeostasis following cellular exposure [10]. However, studies continue to focus on physicochemical antioxidant measurements or cell viability assays rather than investigating intracellular redox signaling. As a result, the extent to which nanobiochar modulates cellular signaling through ROS-dependent mechanisms remains largely unresolved.

### 6.2. Nrf2-Mediated Antioxidant Responses

Among the various redox-sensitive signaling pathways, the nuclear factor erythroid 2-related factor 2 (Nrf2) pathway represents the primary cellular defense mechanism against oxidative stress. Under normal conditions, Nrf2 is retained in the cytoplasm through interaction with Kelch-like ECH-associated protein 1 (Keap1), which promotes its degradation. Exposure to elevated ROS levels induces oxidative modifications within Keap1, resulting in Nrf2 stabilization and nuclear translocation [86]. Once activated, Nrf2 binds to antioxidant response elements (AREs) within target genes and promotes the expression of numerous cytoprotective proteins, including heme oxygenase-1 (HO-1), NAD(P)H quinone oxidoreductase-1 (NQO1), glutathione peroxidase, catalase, and SOD. These antioxidant systems collectively restore redox balance and protect cells from oxidative damage [87].

Studies involving carbon-based nanomaterials suggest that moderate ROS generation at material interfaces may stimulate adaptive antioxidant responses through Nrf2 activation. Similar mechanisms may occur in nanobiochar systems, particularly when incorporated into hydrogels intended for proposed wound healing and tissue regeneration applications [88]. However, this proposed mechanism is inferred primarily from studies on related carbon-based nanomaterials, as direct evidence for Nrf2 activation in nanobiochar–hydrogel systems remains unavailable. Activation of Nrf2 signaling could potentially contribute to protection against oxidative stress and support cellular recovery in damaged tissues [89]. Nevertheless, direct evidence demonstrating Nrf2 activation following nanobiochar exposure remains scarce, and few studies have examined downstream antioxidant gene expression in response to these materials. Consequently, the role of Nrf2 signaling in mediating the therapeutic effects of nanobiochar–hydrogel systems remains largely speculative.

### 6.3. NF-κB and Inflammatory Signaling

The nuclear factor-kappa B (NF-κB) pathway serves as a central regulator of inflammatory and immune responses. Activation of NF-κB occurs in response to numerous stimuli, including cytokines, microbial products, oxidative stress, and cellular injury. ROS can directly influence NF-κB signaling by modifying upstream regulatory proteins and promoting activation of inflammatory transcriptional programs [90].

Upon activation, NF-κB translocates to the nucleus and stimulates the expression of various pro-inflammatory mediators, including tumor necrosis factor-alpha (TNF-α), interleukin-6 (IL-6), interleukin-1β (IL-1β), cyclooxygenase-2 (COX-2), and inducible nitric oxide synthase (iNOS). These molecules coordinate inflammatory responses, recruit immune cells, and influence tissue remodeling processes. While controlled inflammatory signaling is essential for host defense and wound healing, excessive or prolonged activation may contribute to chronic inflammation and tissue damage [91].

The influence of nanobiochar on NF-κB signaling remains poorly characterized. Depending on particle properties, surface chemistry, concentration, and exposure conditions, nanobiochar may either attenuate inflammatory responses through antioxidant activity or promote inflammation through oxidative stress generation. However, most available studies focus on cytotoxicity or antimicrobial activity rather than the direct evaluation of inflammatory signaling pathways. Measurements of inflammatory cytokines, transcription factor activation, and downstream immune responses remain uncommon in the current literature, representing a significant knowledge gap in understanding nanobiochar bioactivity.

### 6.4. MAPK Pathways and Cell Fate Regulation

The mitogen-activated protein kinase (MAPK) signaling network is another major regulator of cellular responses to oxidative stress. The MAPK family includes extracellular signal-regulated kinase (ERK), c-Jun N-terminal kinase (JNK), and p38 MAPK pathways, each of which plays distinct roles in determining cellular fate. These pathways are highly responsive to fluctuations in intracellular ROS levels and participate in regulating proliferation, differentiation, inflammation, stress adaptation, and apoptosis [92,93].

ERK signaling is generally associated with cell survival, proliferation, and tissue regeneration. In contrast, JNK and p38 pathways are more frequently activated under stress conditions and are involved in inflammatory responses and programmed cell death. Excessive ROS generation can stimulate JNK and p38 activation, leading to the expression of stress-related genes and apoptotic signaling cascades [94].

Research involving graphene derivatives, carbon nanotubes, and other carbon-based nanomaterials has demonstrated that material-induced ROS generation can significantly influence MAPK signaling. However, comparable investigations involving nanobiochar remain limited. Consequently, it remains unclear whether nanobiochar promotes regenerative signaling through ERK activation, induces stress responses through JNK and p38 pathways, or exerts concentration-dependent effects involving multiple MAPK branches simultaneously. Understanding these mechanisms will be essential for predicting the biological consequences of nanobiochar exposure in therapeutic settings.

### 6.5. Influence of Nanobiochar on Inflammation, Apoptosis, and Cell Survival

The overall biological response to nanobiochar is likely determined by the balance between adaptive redox signaling and oxidative stress-induced cellular damage. At moderate concentrations, nanobiochar may promote beneficial responses through activation of antioxidant pathways and maintenance of redox homeostasis [95]. Such effects could support cell survival, tissue repair, and regenerative processes. Conversely, excessive ROS generation or prolonged exposure may activate inflammatory signaling pathways, disrupt mitochondrial function, and initiate apoptotic responses [96].

Mitochondria play a central role in these processes because they serve as both sources and targets of intracellular ROS. Elevated oxidative stress can impair mitochondrial membrane integrity, reduce ATP production, and promote the release of cytochrome c into the cytoplasm [97]. This event activates downstream caspase cascades and initiates programmed cell death. Simultaneously, ROS-dependent activation of NF-κB and MAPK pathways can further influence inflammatory signaling and cell fate decisions [98].

These observations highlight the dual nature of nanobiochar-mediated redox activity. Depending on physicochemical properties, dose, exposure duration, and cellular context, nanobiochar may either promote cellular adaptation or trigger pathways associated with toxicity. Distinguishing between these outcomes is particularly important for biomedical applications, where controlled modulation of oxidative stress is desirable, but excessive cellular damage must be avoided. Therefore, mechanistic investigation of signaling pathways should accompany traditional cytocompatibility assessments when evaluating nanobiochar-based therapeutic systems.

In nanobiochar–hydrogel therapeutic systems, the hydrogel matrix may further influence these biological responses by regulating the duration, localization, and concentration of nanobiochar exposure at the tissue interface [45]. By controlling particle mobility, diffusion behavior, and local microenvironmental conditions, hydrogels could potentially support sustained redox activity while reducing excessive cellular exposure. Such characteristics may be advantageous in wound-healing applications, where prolonged regulation of oxidative stress and inflammation is often required to support tissue repair and regeneration [8]. However, direct studies investigating how hydrogel-mediated exposure influences nanobiochar-induced redox signaling responses remain largely unavailable.

### 6.6. Knowledge Gaps in Gene Expression and Signaling Modulation

Despite increasing interest in nanobiochar for biomedical applications, studies directly linking nanobiochar exposure to intracellular signaling events remain remarkably limited. Most investigations focus on physicochemical characterization, antimicrobial activity, antioxidant assays, or short-term viability measurements [2]. While these studies provide useful preliminary information, they offer little insight into the molecular mechanisms governing biological responses.

As a result, it remains unclear whether observed cellular effects arise from activation of adaptive antioxidant pathways, inflammatory signaling networks, mitochondrial stress responses, or apoptotic mechanisms. Furthermore, the potential influence of protein corona formation, hydrogel-mediated exposure, and physiological conditioning on signaling behavior has received minimal attention. This lack of mechanistic understanding represents a major obstacle to the rational design of nanobiochar-based therapeutic systems.

Future studies should incorporate transcriptomic, proteomic, and phosphoproteomic approaches to identify molecular signatures associated with nanobiochar exposure [99]. Techniques such as RT-qPCR, RNA sequencing, Western blotting, ELISA, immunofluorescence imaging, and pathway-specific reporter assays can provide direct evidence of signaling pathway activation and downstream biological responses. Integration of these approaches with intracellular ROS measurements and long-term cellular assessments will be essential for distinguishing beneficial redox modulation from toxicity-associated responses [100].

Collectively, current evidence suggests that nanobiochar possesses the physicochemical characteristics that may enable the modulation of multiple redox-sensitive signaling pathways. However, these proposed biological responses remain largely inferred from related carbon nanomaterials and require direct experimental validation in nanobiochar–hydrogel systems. Bridging this knowledge gap will be critical for advancing nanobiochar–hydrogel systems from promising biomaterials to clinically relevant therapeutic platforms capable of predictable and controlled biological performance.

## 7. Cytocompatibility Assessment and Methodological Limitations in In Vitro Evaluation

Despite increasing interest in nanobiochar-based biomaterials for therapeutic applications, the biological evaluation of these systems remains relatively limited and highly variable [101]. Most studies report cytocompatibility using a small number of viability assays, often under short-term exposure conditions and without detailed investigation of the underlying biological mechanisms [102]. As a result, conclusions regarding safety and therapeutic potential are frequently based on preliminary observations rather than comprehensive biological evidence. Variations in exposure models, dose metrics, assay selection, and experimental duration further complicate comparisons across studies and hinder the development of standardized evaluation frameworks [103]. Given that nanobiochar–hydrogel systems are intended to interact directly with biological tissues, a more rigorous assessment of cytocompatibility is required to establish their safety, functionality, and translational potential.

### 7.1. Exposure Models and Experimental Design

The choice of exposure model significantly influences the interpretation of cytocompatibility results. Current studies commonly employ either extract-based or direct-contact exposure methods. In extract-based models, cells are exposed to media conditioned by the biomaterial, allowing evaluation of soluble components released from the material. These assays are relatively simple to perform and are useful for identifying potentially toxic leachates [104]. However, they do not accurately represent the direct interactions that occur between cells and nanobiochar surfaces.

In contrast, direct-contact models expose cells to the material itself, enabling investigation of particle–cell interactions, membrane responses, cellular uptake, and surface-mediated biological effects. These models more closely resemble physiological exposure conditions but may be influenced by particle aggregation, sedimentation, and variations in local concentration [105]. Consequently, results obtained from extract-based and direct-contact studies are often difficult to compare directly.

Additional complexity arises when nanobiochar is incorporated into hydrogel matrices. Hydrogel networks can regulate particle mobility, diffusion behavior, local concentration gradients, and cell exposure patterns. As a result, biological responses observed in free-particle systems may differ substantially from those observed in nanobiochar–hydrogel composites [106]. Despite this, relatively few studies explicitly compare these exposure scenarios, limiting the current understanding of how hydrogel incorporation influences biological performance.

### 7.2. Dose Metrics and Standardization Challenges

A major challenge in nanobiochar cytocompatibility assessment is the lack of standardized dose normalization strategies. Most studies report exposure concentrations on a mass basis (µg mL^−1^ or mg mL^−1^), assuming that equal mass corresponds to equal biological exposure. However, nanomaterial behavior is strongly influenced by physicochemical characteristics such as particle size, surface area, pore structure, and surface chemistry. Particles with identical mass concentrations may present substantially different biologically active surface areas, resulting in distinct cellular responses. Similarly, aggregation and agglomeration can alter the effective particle number and available surface interactions. Consequently, comparisons among studies using different nanobiochar formulations become difficult when mass concentration is used as the sole exposure metric [107].

Alternative approaches based on particle number concentration or surface area normalization may provide more biologically relevant assessments of exposure. However, these metrics are rarely reported in the current literature. The absence of standardized dose descriptors represents a significant barrier to reproducibility and limits meaningful comparison across studies [107,108]. Establishing harmonized reporting standards will therefore be essential for the future development of nanobiochar-based therapeutic systems.

### 7.3. Limitations of Current Cytocompatibility Endpoints

An additional consideration for nanobiochar-based therapeutic systems is the influence of hydrogel incorporation on cellular exposure. The cytocompatibility profile of free nanobiochar particles may differ substantially from that of nanobiochar embedded within hydrogel matrices because hydrogels regulate particle mobility, diffusion behavior, and the duration of cell–material interactions [109]. Consequently, biological responses observed for isolated nanobiochar cannot necessarily be directly extrapolated to nanobiochar–hydrogel composites, highlighting the need for system-specific biological evaluation [110].

Most published studies evaluate cytocompatibility using conventional viability assays such as MTT, CCK-8, Alamar Blue, neutral red uptake, or live/dead staining [111]. While these methods provide useful preliminary information regarding cellular metabolic activity and membrane integrity, they offer only a limited perspective on biological responses. A common assumption is that high cell viability directly indicates biocompatibility; however, viability measurements alone cannot determine whether cells are experiencing oxidative stress, inflammatory activation, mitochondrial dysfunction, altered gene expression, or disruptions in cellular signaling pathways. Cells may remain metabolically active while simultaneously undergoing significant physiological stress that could compromise long-term functionality, tissue integration, and therapeutic performance [112]. Consequently, viability data alone should not be considered sufficient evidence of biological safety, particularly for materials intended for prolonged biological exposure.

Furthermore, several colorimetric and fluorescence-based assays are susceptible to interference from carbon-based materials. Adsorption of assay reagents onto nanobiochar surfaces, light scattering effects, fluorescence quenching, and interactions with indicator molecules may influence assay outcomes and potentially lead to inaccurate interpretations. Therefore, reliance on a single viability assay may result in an overestimation of material safety [113]. A comprehensive assessment of cytocompatibility should extend beyond simple measurements of cell survival and incorporate complementary endpoints capable of evaluating intracellular ROS generation, mitochondrial function, inflammatory responses, apoptosis, and molecular signaling pathways. Such integrated approaches are essential for establishing the true biological compatibility and therapeutic potential of nanobiochar–hydrogel systems.

### 7.4. Missing Biological Endpoints and Mechanistic Insights

One of the most significant gaps in the current literature is the limited investigation of mechanistic biological responses following nanobiochar exposure. Although oxidative stress regulation is frequently proposed as a key contributor to therapeutic activity, direct measurement of intracellular ROS remains relatively uncommon. Most studies instead rely on cell-free chemical antioxidant assays such as DPPH and ABTS, which measure radical-scavenging activity under simplified experimental conditions and therefore provide only limited insight into intracellular redox regulation and biologically relevant oxidative stress responses [114,115].

Similarly, inflammatory responses are rarely evaluated despite their central importance in tissue repair, immune regulation, and biomaterial acceptance. Measurements of inflammatory cytokines such as TNF-α, IL-6, IL-1β, as well as anti-inflammatory mediators, remain largely absent from many cytocompatibility studies [116]. The influence of nanobiochar on redox-sensitive signaling pathways, including the Nrf2, NF-κB, and MAPK pathways, is also poorly understood. Furthermore, the influence of adsorbed protein layers and protein corona formation on cytocompatibility outcomes remains largely unexplored despite their potential effects on cellular recognition, uptake, intracellular trafficking, and downstream biological responses. Understanding these biointerface-mediated processes will be critical for accurately predicting the biological behavior of nanobiochar-based therapeutic systems.

Mitochondrial function represents another neglected endpoint. Because mitochondria are major sources and targets of intracellular ROS, assessment of mitochondrial membrane potential, respiratory activity, and ATP production could provide valuable insights into cellular responses to nanobiochar exposure [117]. Likewise, studies examining apoptosis, cell-cycle regulation, DNA damage, and cellular senescence remain limited.

Long-term biological responses are particularly underexplored. Most investigations evaluate cellular behavior over periods ranging from 24 to 72 h, whereas therapeutic biomaterials may remain in contact with tissues for weeks or months. Consequently, information regarding prolonged exposure, sustained proliferation, differentiation, tissue integration, and chronic cellular adaptation remains scarce.

Current cytocompatibility assessments provide only a partial understanding of the biological behavior of nanobiochar-based therapeutic systems. Future studies should integrate intracellular ROS measurements, inflammatory biomarkers, mitochondrial function analyses, gene expression profiling, and long-term proliferation assessments to establish a more comprehensive understanding of safety and therapeutic functionality. The limitations of currently employed cytocompatibility assays and the corresponding mechanistic knowledge gaps are summarized in Table 2. These observations highlight the need to move beyond conventional viability-based assessments toward integrated biological evaluation frameworks incorporating oxidative stress, inflammatory signaling, protein corona characterization, advanced culture models, and molecular profiling approaches.

Overall, the current literature demonstrates a strong reliance on short-term viability measurements while providing limited mechanistic understanding of cellular responses to nanobiochar exposure. Addressing these methodological limitations will be essential for establishing reliable safety profiles and supporting the future development of clinically relevant nanobiochar–hydrogel therapeutic systems.

## 8. Behavior in Simulated Physiological Environments

The biological performance of nanobiochar–hydrogel therapeutic systems is strongly influenced by the physicochemical transformations that occur upon exposure to physiological environments. Following implantation or contact with biological fluids, biomaterials are subjected to complex ionic, protein-rich, and dynamic conditions that can alter their surface chemistry, colloidal stability, interfacial interactions, and biological activity. Consequently, the behavior of nanobiochar under physiological conditions cannot be fully predicted from its initial physicochemical characteristics alone. Understanding how these materials evolve in phosphate-buffered saline (PBS), simulated body fluid (SBF), and protein-containing biological media is therefore essential for predicting therapeutic performance, biocompatibility, and long-term functionality.

### 8.1. Stability in PBS and Simulated Body Fluid

Colloidal and structural stability represent critical prerequisites for the biomedical application of nanobiochar-based systems. Physiological media such as PBS and SBF contain elevated ionic strengths that can influence particle aggregation, surface charge, and dispersion behavior [126]. Changes in these parameters directly affect cellular exposure, diffusion within hydrogel matrices, and subsequent biological responses.

Within nanobiochar–hydrogel composites, interactions between biochar surface functionalities and polymer networks contribute significantly to structural stability. Hydroxyl, carboxyl, and other oxygen-containing groups present on biochar surfaces can participate in hydrogen bonding and electrostatic interactions with hydrogel polymers, resulting in enhanced network integrity and reduced polymer chain mobility [9,13]. These interactions influence hydrogel swelling behavior, mesh size, and diffusion characteristics, which ultimately affect nutrient transport, protein infiltration, and therapeutic molecule release.

Several studies have demonstrated that increasing biochar incorporation can strengthen hydrogel networks and reduce equilibrium swelling through enhanced physical crosslinking and structural reinforcement [14,127]. However, most available evidence originates from environmental and agricultural applications rather than physiological systems. Consequently, the stability, aggregation behavior, and structural evolution of nanobiochar–hydrogel composites under biologically relevant ionic conditions remain poorly characterized. Systematic investigations examining particle dispersion, surface charge evolution, and long-term stability in physiological media are still lacking.

### 8.2. Ion Exchange, Mineral Deposition, and Structural Evolution

SBF is widely used to evaluate the bioactivity of biomaterials because its ionic composition resembles that of human plasma. Exposure to SBF frequently initiates ion exchange and mineralization processes that alter material surfaces and influence biological responses. In calcium- and phosphate-containing environments, surface-associated functional groups can serve as nucleation sites for mineral deposition. This process typically begins with adsorption of calcium and phosphate ions, followed by formation of amorphous calcium phosphate and subsequent maturation into hydroxyapatite-like structures [126].

Surface functionalities such as hydroxyl and carboxyl groups are known to facilitate calcium ion binding and promote heterogeneous nucleation, thereby accelerating mineral deposition at biomaterial interfaces [128]. Within hydrogel-based composites, the polymer network further regulates ion diffusion and local supersaturation, creating favorable conditions for controlled mineral growth. Studies involving hydroxyapatite-containing hydrogel systems have demonstrated that polymeric matrices can support gradual apatite formation while maintaining structural integrity under physiological conditions [129].

Although similar mechanisms are likely to occur in nanobiochar-containing composites, direct evidence remains limited. Current understanding suggests that nanobiochar may function as a physicochemical scaffold that influences ion distribution, nucleation density, and interfacial charge characteristics. However, the precise mechanisms governing calcium phosphate deposition, mineral maturation, and structural evolution within nanobiochar–hydrogel systems remain largely unexplored. Advanced in situ characterization techniques will be necessary to clarify these dynamic processes and determine their implications for potential tissue engineering and regenerative medicine applications.

### 8.3. Protein Adsorption and Interfacial Biological Response

Immediately upon exposure to biological fluids, biomaterial surfaces become coated with proteins, forming a dynamic protein layer commonly referred to as the protein corona. This adsorbed protein layer effectively defines the biological identity of the material and strongly influences cellular recognition, adhesion, uptake, and immune responses [130].

Nanobiochar possesses a heterogeneous surface chemistry characterized by aromatic domains, oxygen-containing functional groups, and surface defects that provide multiple binding sites for proteins. Protein adsorption is therefore expected to modify surface charge, wettability, colloidal behavior, and cellular interactions. Previous studies have demonstrated that nanoscale surface properties significantly influence protein adsorption kinetics and the composition of the resulting protein corona [130].

Within hydrogel-based systems, protein adsorption becomes even more complex due to the combined influence of the polymer matrix and embedded nanobiochar particles. The hydrogel network can regulate protein diffusion and accessibility to nanobiochar surfaces, while the nanobiochar component introduces additional adsorption sites capable of selectively interacting with biomolecules. These interactions may influence cell adhesion, macrophage activation, tissue integration, and therapeutic outcomes [131].

However, despite the recognized importance of protein corona formation in nanomedicine, systematic investigations of protein adsorption on nanobiochar–hydrogel systems remain scarce. The composition, temporal evolution, and biological consequences of protein coronas formed on nanobiochar surfaces under physiological conditions have not been comprehensively characterized [132]. This represents a major knowledge gap because protein adsorption may significantly alter cellular responses and potentially influence the redox behavior of nanobiochar-based therapeutic systems.

### 8.4. Influence on Redox Activity and Therapeutic Performance

The redox activity of nanobiochar is closely associated with its surface chemistry, including quinone–hydroquinone moieties, phenolic groups, conjugated carbon structures, and oxygen-containing functional groups. These surface features can participate in reversible electron transfer processes and potentially influence interactions with ROS [10]. Under physiological conditions, however, redox behavior is unlikely to remain static. Protein adsorption, ion exchange, mineral deposition, and structural rearrangements occurring at the biointerface may alter the accessibility and activity of redox-active surface sites. Consequently, the redox properties measured under simplified laboratory conditions may differ substantially from those exhibited in biological environments.

Several studies have suggested that biochar-derived nanomaterials may contribute to oxidative stress regulation through radical-scavenging and electron transfer mechanisms. Nevertheless, direct evidence linking these physicochemical properties to intracellular redox modulation remains limited. Most investigations continue to rely on chemical antioxidant assays rather than biologically relevant measurements of intracellular ROS, mitochondrial function, or redox-sensitive signaling pathways [10,15].

An additional unanswered question concerns the influence of physiological conditioning on redox functionality. Protein corona formation may shield active surface sites, whereas mineral deposition could either inhibit or enhance electron transfer processes. Similarly, incorporation within hydrogel matrices may modify diffusion behavior and alter interactions between nanobiochar surfaces and surrounding cells. Despite the potential importance of these factors, their effects on therapeutic performance remain poorly understood [130].

Collectively, these observations highlight the need to evaluate nanobiochar–hydrogel systems under physiologically relevant conditions rather than relying solely on physicochemical characterization performed in simplified media. Future studies should integrate protein corona analysis, intracellular ROS measurements, mitochondrial assessments, and long-term biological evaluation to establish a mechanistic understanding of how physiological environments influence nanobiochar functionality. Such information will be essential for translating nanobiochar–hydrogel composites from experimental materials into clinically relevant therapeutic systems.

## 9. Toward a Standardized Evaluation Framework

The bibliometric analysis and critical review of the current literature reveal a substantial gap between material development and biological validation in nanobiochar–hydrogel research. While considerable progress has been made in material synthesis, characterization, and environmental applications, the biological evaluation of these systems remains fragmented and inconsistent. Most studies continue to emphasize physicochemical properties and simple antioxidant measurements, whereas mechanistic investigations of cellular responses remain limited. Similar challenges have been identified across the broader fields of nanomedicine and biomaterials, where insufficient standardization has hindered reproducibility, comparability, and clinical translation [133,134]. Therefore, a structured evaluation framework is needed to facilitate the development of clinically relevant nanobiochar–hydrogel therapeutic systems.

### 9.1. Physiologically Relevant Testing Conditions

A major limitation of current studies is the reliance on simplified experimental environments that do not adequately represent biological conditions. Since nanobiochar surface properties can be altered by proteins, ions, and other biomolecules, future studies should routinely evaluate materials in physiologically relevant media such as PBS, SBF, and serum-containing culture systems. These conditions provide a more realistic understanding of material stability, aggregation behavior, protein adsorption, and redox activity. Characterization under biologically relevant conditions is increasingly recognized as an essential component of nanomaterial safety and performance assessment [133,134].

An additional consideration is the distinction between free nanobiochar particles and nanobiochar incorporated within hydrogel matrices. Because hydrogels can modify particle mobility, diffusion behavior, protein interactions, and cellular exposure profiles, biological responses observed for isolated nanobiochar may not accurately predict the behavior of nanobiochar–hydrogel composites. Therefore, future evaluation frameworks should assess both free nanobiochar and nanobiochar–hydrogel formulations to establish the contribution of each component to overall biological performance. The major knowledge gaps identified throughout the current literature and the corresponding priorities for future biological evaluation of nanobiochar–hydrogel therapeutic systems are summarized in Table 3.

### 9.2. Mechanistic Evaluation of Redox Activity

The antioxidant potential of nanobiochar is frequently assessed using DPPH and ABTS assays. Although useful for preliminary screening, these methods provide limited information regarding biological function. Future evaluation frameworks should prioritize intracellular ROS measurements, mitochondrial oxidative stress assessment, and antioxidant defense responses [115]. Techniques such as DCFH-DA, MitoSOX, glutathione quantification, and antioxidant enzyme analysis can provide a more comprehensive understanding of how nanobiochar influences cellular redox homeostasis [10]. Such approaches would help distinguish simple chemical radical scavenging from biologically relevant redox modulation [139].

### 9.3. Integration of Biological Response Pathways

ROS influence numerous cellular processes beyond oxidative stress. Therefore, future studies should extend biological evaluation to include signaling pathways associated with inflammation, stress adaptation, and cell survival [140]. Particular emphasis should be placed on pathways such as Nrf2, NF-κB, and MAPK, which are central regulators of redox biology [136]. In addition, inflammatory biomarkers, including TNF-α, IL-1β, IL-6, and IL-8, should be incorporated into routine assessment protocols. Combining ROS measurements with pathway analysis and cytokine profiling would provide a mechanistic understanding of nanobiochar–cell interactions rather than relying solely on viability measurements [116].

### 9.4. Long-Term and Application-Specific Biological Assessment

Many existing studies evaluate biological responses over short exposure periods, typically 24–72 h. However, biomedical applications such as wound dressings, tissue engineering scaffolds, and implantable materials involve prolonged interaction with biological tissues. Future studies should therefore include long-term cytocompatibility assessments, repeated-dose exposure models, and monitoring of cellular adaptation over time. Where appropriate, advanced models such as three-dimensional cultures, co-culture systems, microfluidic platforms, and organ-on-chip technologies may provide additional mechanistic insight and improve physiological relevance [137,138].

### 9.5. Standardization and Reproducibility

A significant challenge in nanobiochar research arises from variability in biomass feedstock, pyrolysis conditions, activation procedures, and post-processing methods. These factors strongly influence surface chemistry, defect density, functional groups, and biological activity. Consequently, future studies should adopt standardized reporting practices that include detailed information on biomass sources, production parameters, physicochemical characterization, and biological testing conditions. Similar recommendations have been proposed throughout the nanomedicine field to improve reproducibility and facilitate cross-study comparisons [133,134]. Establishing harmonized characterization and reporting protocols will be essential for accelerating the biomedical translation of nanobiochar-based materials.

Overall, future progress in nanobiochar–hydrogel research will depend on moving beyond descriptive material characterization toward integrated biological evaluation. A framework that combines physiologically relevant testing, intracellular redox assessment, signaling pathway analysis, inflammatory profiling, long-term cytocompatibility studies, and standardized reporting practices will provide a stronger foundation for understanding biological performance and advancing the development of clinically relevant therapeutic systems.

## 10. Conclusions

Nanobiochar–hydrogel systems have attracted increasing interest as potential therapeutic biomaterials due to the complementary properties of both components. While hydrogels provide a hydrated three-dimensional environment capable of supporting localized delivery, tissue integration, and wound management, nanobiochar offers unique physicochemical characteristics, including redox-active surface functionalities, high surface area, adsorption capacity, and potential antimicrobial activity. Together, these features suggest a promising platform for biomedical applications; however, the biological mechanisms governing their interactions remain poorly understood.

The findings of this review reveal a substantial gap between material development and biological validation. Bibliometric analysis demonstrated that biochar–hydrogel research remains largely focused on environmental remediation, adsorption processes, and material characterization, with comparatively limited attention given to therapeutic applications. Consequently, many assumptions regarding the biological performance of nanobiochar are currently based on physicochemical properties rather than direct mechanistic evidence. Furthermore, biological evaluations frequently rely on chemical antioxidant assays and short-term viability measurements, providing limited insight into intracellular ROS regulation, inflammatory responses, mitochondrial function, cellular uptake, protein corona formation, and signaling pathway activation.

Particular attention should be directed toward understanding how nanobiochar influences redox-sensitive biological pathways involved in oxidative stress regulation, inflammation, apoptosis, and tissue repair. The limited availability of studies examining Nrf2, NF-κB, MAPK signaling, and gene expression responses highlights a critical barrier to the rational development of nanobiochar-based therapeutic systems. Likewise, the effects of physiological conditioning, protein adsorption, hydrogel-mediated exposure, and long-term cellular interactions remain largely unexplored.

From a wound-healing perspective, the combination of hydrogel-based structural support with the potential redox-modulating and antimicrobial properties of nanobiochar provides a compelling scientific rationale for further investigation. However, direct biological evidence supporting these functions remains scarce, emphasizing the need for systematic and standardized evaluation. The most urgent research priorities include direct intracellular ROS quantification, protein corona characterization, long-term cytocompatibility assessment, biofilm-specific evaluation, inflammatory and immune response profiling, mitochondrial function analysis, investigation of hydrogel-mediated exposure, and testing under physiologically relevant conditions. Addressing these critical gaps through integrated biological, molecular, and physiologically relevant in vitro studies will be essential for establishing clear relationships between nanobiochar surface chemistry, biointerface interactions, cellular signaling, and therapeutic performance. Such evidence will determine whether nanobiochar–hydrogel systems can progress from promising biomaterial candidates to clinically relevant platforms for wound healing and regenerative medicine.

## Figures and Tables

**Table 1:** Representative biochar/nanobiochar–hydrogel systems and related studies highlighting the current evidence base, biological evaluation, and knowledge gaps for therapeutic applications.

Biochar Source	Synthesis/ Pyrolysis Conditions	Particle Size & Surface Chemistry	Hydrogel Matrix	Intended Application	Biological Model	Biological Evaluation	Main Limitations
Cow dung	Pyrolyzed under N_2_	Porous carbon particles; rough surface; photothermal- responsive	N-Halamine hydrogel	Infected wound healing	Wound infection models	Multimodal antibacterial and wound healing evaluation	Lacks intra- cellular signaling analysis [8].
Local Egyptian plant biomass	Not explicitly stated	Specific surface area ~ 134 m^2^ g^−1^. mesoporous structure	Gellan Gum/ Poly vinyl alcohol) PVA Hydrogel membrane	Water pollutant remediation	None (acellular)	None	Environmental adsorption study; no compatibility evaluation [9].
Wheat straw derived biochar	Pyrolysis under N_2_ atmosphere at 400 °C and 700 °C	Nano-sized (78–122 nm); oxygen functional groups	None	Environmental redox mediator for Fe/Mn reduction and dye degradation	None (acellular)	None	Simplified aqueous testing; lacks physiological and hydrogel integration [10].
Phoenix Dactylifera (date palm) biomass-derived biochar	Prepared as an nZVCe/biochar composite	Mesoporous nZVCe/BC; Ce dispersed on biochar	None	Environmental remediation and basic bioactivity	Planktonic micro- organisms	None	Planktonic models only; no biofilm evaluation [11].
Agricultural stalk-derived biochar	Not explicitly stated	Heterointerfaces optimized for electron transfer	Biochar-modified (PVA)/gelatin/quaternized chitosan hydrogel	Wastewater resource recovery & nutrient utilization	None (acellular system)	None	Environmental application only; no biological or therapeutic evaluation [12].
Agricultural stalk-derived biochar	Pyrolysis of agricultural stalks at 750 °C.	0.49–0.72 nm crystallites; 200–300 nm hydrogel coating; active binding sites	Biochar-modified PVA/gelatin/QCS hydrogel	Controlled nutrient release from wastewater-recovered nutrients	None (acellular)	None	Environmental/ agricultural application only; no biomedical evaluation [13].
Vine pruning waste	500 °C slow pyrolysis; <200 µm biochar	Honeycomb porous structure (micro/meso/ macropores); OH, C=O, C–O–C, aromatic groups	CaCl_2_-cross-linked biochar/PVA–SA hydrogel	Agricultural water retention	Plant seed model	None	Agricultural evaluation only; no biomedical safety assessment [14].
ZnCl_2_-activated Fe_3_O_4_-magnetized licorice biochar	ZnCl_2_ activation with Fe_3_O_4_ nanoparticle incorporation	Mesoporous magnetic biochar/Fe_3_O_4_ composite; O-functional groups	None	Multifunctional dye adsorbent with antimicrobial properties	Bacteria, Fungal strains, and erythrocytes	None	Antimicrobial activity only; no cytocompatibility or therapeutic evaluation [15].

**Table 2:** Current in vitro evaluation approaches and mechanistic gaps in nanobiochar–hydrogel systems.

Model/Assay	Endpoint	Critical Limitations	Missing Mechanistic Insight
Extract-based (ISO 10993-5): MTT, MTS, WST 1	Metabolic activity (%)	Tetrazolium reagent adsorption onto carbon surfaces produces systematic false-negative viability readings (all variants affected)	Membrane disruption, NF-κB/Nrf2 activation, ROS signaling, and inflammatory cytokine release [118].
Direct-contact: Live/Dead (Calcein-AM/EthD-1)	Membrane integrity (%)	Fluorescence quenching and particle aggregation generate false negatives; fails to capture sub-lethal toxicity.	Apoptotic pathways, mitochondrial dysfunction, and sub-lethal oxidative stress [119].
Direct-contact: Trypan blue exclusion	Cell viability (%)	Detects only late-stage membrane damage; cannot distinguish apoptosis from necrosis; operator-dependent	Masks intracellular signaling [120].
Acellular ROS: DCFH-DA	ROS generation (RFU)	Probe auto-oxidation; particle-type-specific reproducibility errors	Direct redox indication and intracellular signaling [121].
Intracellular ROS: DCFH	ROS generation	Inability of plate readers to differentiate intra and extracellular ROS. Potential overestimation of oxidative stress	Lacks mechanistic insight into ROS generation pathways [122].
Organ-on-a-chip (microfluidic)	TEER, cell viability Tight-junction (TJ) protein expression	Insufficient standardization; no validated nanobiochar-specific protocols	Protein corona kinetics, inflammatory signaling, and chronic-dose response [123].
Macrophage/immune models (RAW264.7, THP-1)	Inflamasome activation (NLRP3 assembly), inflammatory cytokine release (IL-1β)	Simplified immune environment; high donor-to-donor variability in primary cells	ROS involvement, NF-κB/MAPK signaling, M1/M2 polarization [124].
Dynamic exposure (Air–Liquid Interface (ALI) co-culture)	Nanoparticle deposition efficiency, ROS generation	Limited standardization of flow rate and shear stress conditions	Does not fully resolve whole-organ immune regulation or multi-cellular lung microenvironment complexity [39].
In vivo wound healing models	Histology, IHC, biodistribution	Ethical constraints; interspecies differences; high cost and throughput limitations	Systemic immune response, biodistribution, clearance, and long-term toxicity [125].

**Table 3:** Current knowledge gaps and future research priorities for the biological evaluation of nanobiochar–hydrogel therapeutic systems.

Research Area	Current Knowledge	Major Limitation	Recommended Future Investigation
Surface redox chemistry	Electron transfer and radical-scavenging properties demonstrated mainly in environmental systems	Limited validation under physiological conditions	Evaluate redox behavior in biologically relevant media [10,15].
Intracellular ROS regulation	Mostly inferred from DPPH and ABTS assays	Lack of intracellular ROS measurements	Quantify ROS using DCFH-DA, MitoSOX, and related assays [115].
Protein corona formation	Well established for nanomaterials in general	Virtually unexplored for nanobiochar	Characterize corona composition using proteomics [130,131].
Cellular uptake	Uptake pathways known for other carbon nanomaterials	Limited data for nanobiochar	Investigate uptake mechanisms and intracellular fate [135].
Signaling pathways	Nrf2, NF-κB, and MAPK implicated in oxidative stress responses	Direct evidence in nanobiochar systems is scarce	Integrate gene expression and pathway analyses [136].
Cytocompatibility assessment	Predominantly short-term viability assays	Lack of mechanistic and long-term endpoints	Include inflammation, mitochondrial function, and apoptosis markers [111].
Hydrogel-integrated systems	Emerging research area	Limited biological evaluation	Assess hydrogel-mediated modulation of biointerface interactions [109,110].
Physiological conditioning	Few studies in PBS, SBF, or serum-containing media	Poor understanding of surface evolution and stability	Evaluate protein adsorption, mineralization, and long-term transformations [126,133,134].
Advanced biological models	Primarily 2D cell culture studies	Limited physiological relevance	Employ 3D cultures, organ-on-chip systems, and co-culture models [137,138].

## Data Availability

No new data were created or analyzed in this study. Data sharing is not applicable to this article.

## Abbreviations

The following abbreviations are used in this manuscript:

ABTS	2,2’-Azino-bis (3-ethylbenzothiazoline-6-sulfonic acid)
AFM	Atomic Force Microscopy
ALI	Air–Liquid Interface
ARE	Antioxidant Response Element
ATP	Adenosine Triphosphate
BC	Biochar
CAM	Calcein acetoxymethyl ester
CAT	catalase
CCK-8	Cell Counting Kit-8
COOH	Carboxyl Group
COX-2	Cyclooxygenase-2
DCFH-DA	2’,7’-Dichlorofluorescin Diacetate
DNA	Deoxyribonucleic Acid
DPPH	2,2-Diphenyl-1-picrylhydrazyl
EFSA	European Food Safety Authority
ELISA	Enzyme-Linked Immunosorbent Assay
ERK	Extracellular Signal-Regulated Kinase
FTIR	Fourier Transform Infrared Spectroscopy
Fe	Iron
Fe_3_O_4_	Magnetite (Iron Oxide) Nanoparticles
GPx	Glutathione peroxidase
GQP	Gelatin-Quaternized Chitosan–Poly (vinyl alcohol)
GSH	Glutathione
H_2_O_2_	Hydrogen Peroxide
HO-1	Heme Oxygenase-1
•OH	Hydroxyl Radical
IHC	Immunohistochemistry
IL-1β	Interleukin-1 Beta
IL-6	Interleukin-6
IL-8	Interleukin-8
iNOS	Inducible Nitric Oxide Synthase
ISO	International Organization for Standardization
JNK	c-Jun N-terminal Kinase
Keap1	Kelch-like ECH-Associated Protein 1
MAPK	Mitogen-Activated Protein Kinase
MDA	Malondialdehyde
MTS	3-(4,5-dimethylthiazol-2-yl)-5-(3-carboxymethoxyphenyl)-2-(4- sulfophenyl)-2H-tetrazolium
MTT	3-(4,5-dimethylthiazol-2-yl)-2,5-diphenyltetrazolium Bromide
MWCNTs	Multiwalled carbon nanotubes
NF-κB	Nuclear Factor-Kappa B
NLRP3	NOD-like Receptor Protein 3
NQO1	NAD(P)H Quinone Oxidoreductase-1
Nrf2	Nuclear Factor Erythroid 2-Related Factor 2
NZVCe	Nano Zero-Valent Cerium
O_2_•^−^	Superoxide Radical
p38	p38 Mitogen-Activated Protein Kinase
PBS	Phosphate-Buffered Saline
pH	Potential of Hydrogen
PI	Propidium Iodide
PVA	Poly (vinyl alcohol)
RFU	Relative Fluorescence Units
RNA	Ribonucleic Acid
ROS	Reactive Oxygen Species
RT-qPCR	Reverse Transcription Quantitative Polymerase Chain Reaction
SBF	Simulated Body Fluid
SEM	Scanning Electron Microscopy
SOD	Superoxide Dismutase
TEER	Transepithelial Electrical Resistance
TEM	Transmission Electron Microscopy
TMA-DPH	Trimethylammonium-diphenylhexatriene
TNF-α	Tumor Necrosis Factor-Alpha
WST-1	Water-Soluble Tetrazolium-1
XRD	X-Ray Diffraction
XTT	2,3-bis(2-methoxy-4-nitro-5-sulfophenyl)-2H-tetrazolium-5-carboxanilide
ZnCl_2_	Zinc Chloride
ZOI	zone-of-inhibition
